# Genetic Evidence for the Causal Relationship Between Gut Microbiota and Diabetic Kidney Disease: A Bidirectional, Two-Sample Mendelian Randomisation Study

**DOI:** 10.1155/2024/4545595

**Published:** 2024-10-23

**Authors:** Yun Zhang, Lingyun Zhao, Yifan Jia, Xin Zhang, Yueying Han, Ping Lu, Huijuan Yuan

**Affiliations:** ^1^Department of Endocrinology, Henan Provincial People's Hospital & People's Hospital of Zhengzhou University & People's Hospital of Henan University, Zhengzhou, Henan, China; ^2^Xinxiang Medical University, Xinxiang, Henan, China; ^3^Department of Endocrinology, People's Hospital of Zhengzhou University, Zhengzhou, Henan, China

**Keywords:** diabetes, diabetic kidney disease, gut, gut microbiota, kidney axis, Mendelian randomisation

## Abstract

**Aims:** According to the gut–kidney axis theory, gut microbiota (GM) has bidirectional crosstalk with the development of diabetic kidney disease (DKD). However, empirical results have been inconsistent, and the causal associations remain unclear. This study was aimed at exploring the causal relationship between GM and DKD as well as the glomerular filtration rate (GFR) and urinary albumin-to-creatinine ratio (UACR).

**Materials and Methods:** Two-sample Mendelian randomisation (MR) analysis was performed with inverse-variance weighting as the primary method, together with four additional modes (MR–Egger regression, simple mode, weighted mode, and weighted median). We utilised summary-level genome-wide association study statistics from public databases for this MR analysis. Genetic associations with DKD were downloaded from the IEU Open GWAS project or CKDGen consortium, and associations with GM (196 taxa from five levels) were downloaded from the MiBioGen repository.

**Results:** In forward MR analysis, we identified 13 taxa associated with DKD, most of which were duplicated in Type 2 diabetes with renal complications but not in Type 1 diabetes. We observed a causal association between genetic signature contributing to the relative abundance of Erysipelotrichaceae UCG003 and that for both DKD and GFR. Similarly, host genetic signature defining the abundance of Ruminococcaceae UCG014 was found to be simultaneously associated with DKD and UACR. In reverse MR analysis, the abundance of 14 other GM taxa was affected by DKD, including the phylum Proteobacteria, which remained significant after false discovery rate correction. Sensitivity analyses revealed no evidence of outliers, heterogeneity, or horizontal pleiotropy.

**Conclusion:** Our findings provide compelling causal genetic evidence for the bidirectional crosstalk between specific GM taxa and DKD development, contributing valuable insights for a comprehensive understanding of the pathological mechanisms of DKD and highlighting the possibility of prevention and management of DKD by targeting GM.

## 1. Introduction

Approximately 40% of patients with diabetes clinically develop diabetic kidney disease (DKD), one of the most common and severe microvascular complications, also known as diabetic nephropathy. DKD is characterised by increased proteinuria and decreased glomerular filtration rate (GFR) [1] and is a major global health burden as the primary origin of chronic kidney disease and end-stage kidney disease. Moreover, it is independently associated with cardiac events and all-cause mortality [2, 3]. While intensive efforts to control hyperglycaemia, hypertension, and dyslipidaemia can slow DKD progression, the percentage of patients progressing to end-stage kidney disease continues to rise, highlighting the need for sustainable treatment [4]. Thus, the identification of novel potential pathogenic factors and therapeutic strategies for DKD is urgently needed.

Recently, the gut–kidney axis has gained considerable attention owing to accumulating evidence on the role of gut microbiota (GM) in the pathogenesis of DKD. The GM consists of approximately 10^13^–10^14^ microorganisms (bacteria, fungi, viruses, and other microbial species) residing in the intestinal tract, 98% of which are bacteria [5]. Dysbiosis of the GM has been linked to nearly 95% of all health conditions, particularly metabolic diseases such as diabetes [6]. Several observational studies have revealed distinctions in the GM of patients with DKD compared with that of healthy individuals, including a decrease in the abundance of probiotics such as *Intestinibacter* and *Lachnospira* and an enrichment of pernicious bacteria such as *Coprobacillus* and *Desulfovibrio* [7–9]. Additionally, increased proportion of *Escherichia*–*Shigella* and reduced proportion of *Prevotella_9* have been found in stool samples of patients with DKD compared with those of individuals with diabetes [10]. Microbiota-associated metabolites such as trimethylamine N-oxide (TMAO) and short-chain fatty acids (SCFAs) are also involved in DKD development [11, 12]. However, the results have not been consistent across studies. For example, Du et al. [13] reported that the richness and diversity of the GM in patients with DKD were significantly decreased compared to those in healthy individuals, whereas Zhang et al. [9] did not observe any changes in the diversity of GM in the DKD group compared with that in the diabetes or healthy control groups.

Inconsistent findings in traditional observational studies may have resulted from inherent issues such as reverse causation, selection bias, and environmental confounders. Additionally, observational studies cannot establish causality. Recently, Mendelian randomisation (MR) has offered a novel approach to access the causal effect of risk factors on disease development by integrating genomic data into conventional observational studies [14, 15]. By relying on genetic variants being passed down randomly at conception, reverse causality and environmental bias are minimised. In this study, the causal link between host genetic components contributing to the relative abundance of each GM taxa and DKD development was explored through a bidirectional two-sample MR analysis.

## 2. Materials and Methods

### 2.1. Study Design

**Figure 1:**
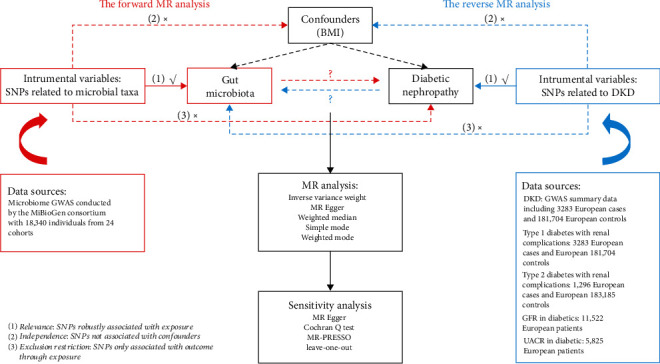
Flowchart of the bidirectional MR study to explore the causal crosstalk between GM and diabetic nephropathy development. BMI: body mass index; DKD: diabetic kidney disease; GFR: glomerular filtration rate; GM: gut microbiota; GWAS: genome-wide association study; MR: Mendelian randomisation; SNPs: single-nucleotide polymorphisms; UACR: urinary albumin-to-creatinine ratio.

Figure 1 displays the methodology flowchart of our study. We utilised summary-level genome-wide association study (GWAS) statistics from public databases for this MR analysis. Initially, the study established GM as the exposure and DKD as the outcome in a forward MR analysis to assess the role of GM in promoting or preventing DKD development. To investigate the changes in the GM among patients with DKD, we performed a reverse MR analysis, with GM as the outcome and DKD as the exposure. The STROBE-MR guidelines (https://www.strobe-mr.org/) [16, 17] were followed for both forward and reverse MR analyses. The details are listed in Supporting Information 5: Table S1.

### 2.2. Data Sources

For single-nucleotide polymorphisms (SNPs) associated with human GM, we downloaded the summary-level data from a meta-analysis of 24 GWAS cohorts focusing on GM conducted by the MiBioGen team. The study involved 18,340 individuals mainly from Europe. The microbiota compositions of human faecal samples were assessed using 16S rRNA sequencing and classified into five categories: 9 phyla, 16 classes, 20 orders, 35 families, and 131 genera, excluding 12 unknown genera and 3 unknown families. More detailed information is available in the literature [18, 19].

For DKD, GWAS summary data of 3283 European cases and 181,704 European controls were extracted from the IEU Open GWAS project. The ICD-10 (code: N08.3⁣^∗^) criterion was used as the definition for DKD in this cohort. We also extracted GWAS data on Type 1 (1206 cases and 183,185 controls) and Type 2 (963 cases and 183,185 controls) diabetes with renal complications from the FinnGen biobank. All of the participants in these cohorts were Europeans. From the CKDGen consortium, we obtained GWAS summary-level data on urinary albumin-to-creatinine ratio (UACR) of 5825 European patients with diabetes [20]. GWAS summary data for GFR in individuals with diabetes were downloaded from another IEU Open GWAS project (GWAS ID ebi-a-GCST003373), which included 11,522 European individuals [21]. The details are listed in Supporting Information 5: Table S2.

### 2.3. Selection of Eligible Instrumental Variables (IVs)

SNPs were identified as eligible IVs in both forward and reverse MR analyses with the same criteria: (1) SNPs must be strongly related to the levels of exposure (a genome-wide significant threshold of *p* < 1 × 10^5^ as per previous studies); (2) SNPs must be independent, defined as having a low linkage disequilibrium threshold (*r*^2^ < 0.001, kb = 10,000) in the European population; and (3) significant associations with the outcome were excluded to ensure that SNPs were related to outcomes only by way of exposure. The PhenoScanner tool [22] was used to assess and exclude the effects of confounders, such as age, sex, smoking, and body mass index. *F* statistic was computed, and IVs with high *F* statistics (> 10) were extracted.

### 2.4. Statistical Methods and Sensitivity Analysis

The TwoSampleMR package (0.5.7) in R was used for all MR-related analyses. We used five methods to explore the causative linkages between exposure and outcome, with the inverse-variance-weighted (IVW) mode as the major method and four other modes (MR–Egger regression, simple mode, weighted mode, and weighted median) as supplementary methods.

The heterogeneity among the various causal effects was assessed with Cochran's *Q* statistic. If heterogeneity existed with *p* < 0.05, the random-effect mode of the IVW was employed. MR–Egger regression was utilised to explore pleiotropy. The “leave-one-out” analysis was also employed for sensitivity analysis. Outliers were explored using the global test of MR-PRESSO.

Results of our MR analyses are displayed as odds ratio (OR) together with corresponding 95% confidence interval (CI). Results with *p* < 0.05 were defined as significant, and then, the *p* value was adjusted by false discovery rate (FDR) correction. A *p* < 0.05 without correction (*p*‐FDR ≥ 0.05) signified a nominal association, whereas *p*‐FDR < 0.05 indicated a significant causal association. All analyses were performed in R 4.3.1.

### 2.5. Ethical Approval

We used summary-level GWAS data from public databases for the MR analyses in our study. No ethical approval was needed.

## 3. Results

### 3.1. Causal Effect of GM on DKD Development

**Figure 2:**
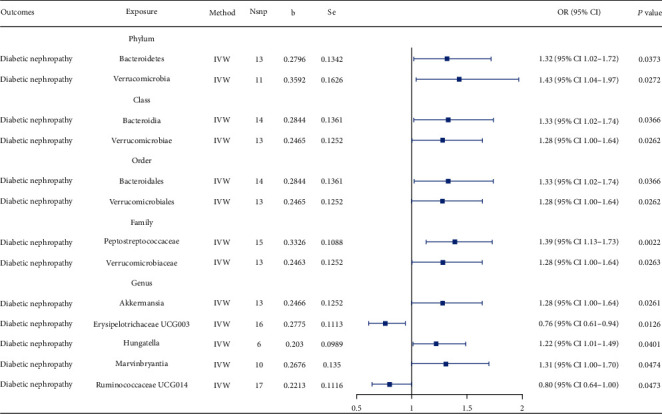
MR-estimated causal effect of gut microbiota and diabetic kidney disease development using the IVW statistical model. IVW: inverse-variance weighted; Nsnp: number of single-nucleotide polymorphisms selected as instrumental variables.

We identified potential IVs for GM phenotypes based on the selection criteria. The range of SNPs for each tested GM phenotype was 4–26 (Supporting Information 5: Table S3). Using the IVW method, we initially identified 13 bacterial taxa causally associated with DKD development (Figure 2; Supporting Information 5: Table S4). At the phylum level, both Bacteroidetes (OR = 1.32, 95% CI 1.02–1.72, *p* = 0.0373) and Verrucomicrobia (OR = 1.43, 95% CI 1.04–1.97, *p* = 0.0272) were found to nominally associate with increased risk of DKD. For Bacteroidetes, Bacteroidia at the class level (OR = 1.33, 95% CI 1.02–1.74, *p* = 0.0366) and Bacteroidales at the order level (OR = 1.33, 95% CI 1.02–1.74, *p* = 0.0366) had causal effects on DKD. For Verrucomicrobia, Verrucomicrobiae at the class level (OR = 1.28, 95% CI 1.00–1.64, *p* = 0.062), Verrucomicrobiales at the order level (OR = 1.28, 95% CI 1.00–1.64, *p* = 0.0262), Verrucomicrobiaceae at the family level (OR = 1.28, 95% CI 1.00–1.64, *p* = 0.0263), and *Akkermansia* at the genus level (OR = 1.28, 95% CI 1.00–1.64, *p* = 0.0261) were causally associated with DKD development. For the phylum Bacillota, one taxa at the family level (Peptostreptococcaceae, OR = 1.39, 95% CI 1.13–1.73, *p* = 0.0022) and two taxa at the genus level (*Hungatella*, OR = 1.22, 95% CI 1.01–1.49, *p* = 0.0401; *Marvinbryantia*OR = 1.31, 95% CI 1.00–1.70, *p* = 0.0474) were associated with an increased risk of DKD, whereas Erysipelotrichaceae UCG003 (OR = 0.76, 95% CI 0.61–0.94, *p* = 0.0126) and Ruminococcaceae UCG014 (OR = 0.80, 95% CI 0.64–1.00, *p* = 0.0473) showed a protective effect against DKD development. However, none of them remained significantly associated with DKD development after FDR correction. All four supplementary MR methods revealed the same direction of influence as IVW, except for all four taxa belonging to the phylum Verrucomicrobia from the class to genus levels (class Verrucomicrobiae, order Verrucomicrobiales, family Verrucomicrobiaceae, and genus *Akkermansia*) in the MR–Egger mode (Supporting Information 1: Figure S1).

For a more in-depth analysis of the effect of GM on DKD development, we analysed the renal complications in two types of diabetes separately. Most causal relationships identified above (10/13) were validated in Type 2 diabetes with renal complications. None of them were significant in Type 1 diabetes with renal complications.

Increased proteinuria and decreased GFR are the clinical characteristics of DKD. We also explored the causal effect of the GM on the UACR and GFR in patients with diabetes. For UACR, Ruminococcaceae UCG014 (OR = 1.53, 95% CI 1.02–2.29, *p* = 0.0394) was initially identified to be causally associated with the UACR through IVW, together with three other taxa that were not significant in the MR analysis of GM and DKD. For GFR, Erysipelotrichaceae UCG003 (OR = 0.93, 95% CI 0.87–0.99, *p* = 0.0474) was initially identified to be causally linked with GFR by IVW, together with six other taxa that were not significant in the MR analysis of GM and DKD (Supporting Information 5: Table S5).

### 3.2. Causal Effect of DKD on GM

**Figure 3:**
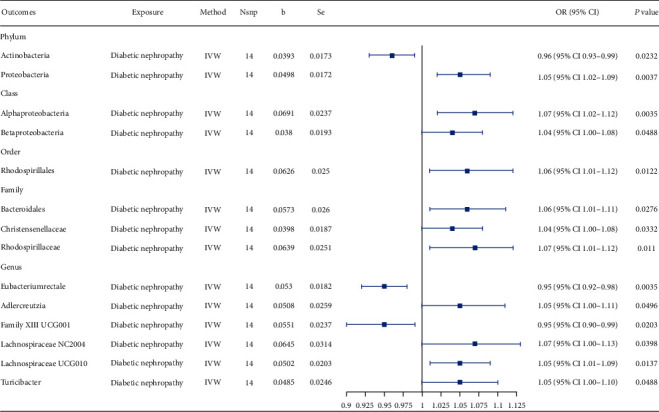
MR-estimated causal effect of diabetic kidney disease on the gut microbiota using the IVW statistical model. IVW: inverse-variance weighted; Nsnp: number of single-nucleotide polymorphisms selected as instrumental variables.

For DKD as the exposure, 25 SNPs were selected as IVs (Supporting Information 5: Table S6), and none of them were related to potential confounders in the PhenoScanner tool. Fourteen bacterial taxa were initially identified to be causally associated with the GM by IVW (Figure 3; Supporting Information 5: Table S7). For the phylum Actinobacteria, decreased proportions of Actinobacteria species at the phylum level (OR = 0.96, 95% CI 0.93–0.99, *p* = 0.0232) and increased proportions of *Adlercreutzia* species at the genus level (OR = 1.05, 95% CI 1.00–1.11, *p* = 0.0496) were found in patients with DKD. For the phylum Proteobacteria, Proteobacteria at the phylum level (OR = 1.05, 95% CI 1.02–1.09, *p* = 0.0037), Alphaproteobacteria (OR = 1.07, 95% CI 1.02–1.12, *p* = 0.0035) and Betaproteobacteria (OR = 1.04, 95% CI 1.00–1.08, *p* = 0.0488) at the class level, Rhodospirillales at the order level (OR = 1.06, 95% CI 1.01–1.12, *p* = 0.0122), and Rhodospirillaceae at the family level (OR = 1.07, 95% CI 1.01–1.12, *p* = 0.0110) were positively associated with DKD. For the phylum Bacillota, one taxa at the family level (Christensenellaceae, OR = 1.04, 95% CI 1.00–1.08, *p* = 0.0332) and three taxa at the genus level (Lachnospiraceae NC2004, OR = 1.07, 95% CI 1.00–1.13, *p* = 0.0398; Lachnospiraceae UCG010, OR = 1.05, 95% CI 1.01–1.09, *p* = 0.0137; and *Turicibacter*, OR = 1.05, 95% CI 1.00–1.10, *p* = 0.0488) showed a positive association with DKD development, while one taxa at the genus level (*Eubacterium rectale* group, OR = 0.95, 95% CI 0.92–0.98, *p* = 0.0035) showed a negative association with DKD development. In addition, the proportion of the family Bacteroidales S24.7 group belonging to the phylum Bacteroidota (OR = 1.06, 95% CI 1.01–1.11, *p* = 0.0276) and that of the genus Family XIII UCG001 belonging to the phylum Firmicutes (OR = 0.95, 95% CI 0.90–0.99, *p* = 0.0203) were also nominally influenced by DKD. Only the effect of DKD on the proportion of Proteobacteria species remained significant after FDR correction (*p*‐FDR = 0.0185). All four supplementary MR methods revealed the same direction of influence as IVW, except for Rhodospirillales members at the order and Rhodospirillaceae members at the family level in the simple mode analysis (Supporting Information 2: Figure S2).

### 3.3. Sensitivity Analysis

In both forward and reverse MR analyses, we did not identify any indication of outliers, heterogeneity, or horizontal pleiotropy in MR-PRESSO, Cochran's *Q* test, or MR–Egger regression (Supporting Information 5: Tables S4 and S7). The “leave-one-out” analysis showed that the results were not significantly changed by the removal of any single SNP (Supporting Information 3: Figure S3 and Supporting Information 4: Figure S4). All these results confirmed the robustness and reliability of our MR analysis.

## 4. Discussion

To the best of our knowledge, this is the first study to utilise MR analysis to explore the causal crosstalk between the GM and DKD development. We used publicly available GWAS data with large cohorts and confirmed a bidirectional interaction between GM and DKD. Genetic signatures contributing to the relative abundance of some specified GM taxa causally associated with DKD were different between Type 1 and Type 2 diabetes. Moreover, our study introduces novel evidence indicating a potential causal effect of host genetic components contributing to several GM taxa on the UACR and GFR.

Accumulating evidence has unveiled a bidirectional crosstalk between the gastrointestinal tract and kidneys, which is defined as the gut–kidney axis. In essence, pathophysiological alterations in the kidneys or gastrointestinal tract may affect or impair the other side [23]. Herein, we provide genetic causal evidence for the bidirectional interaction between specific GM taxa and DKD development from MR analyses. In brief, 11 GM taxa contributed to a high risk of DKD, whereas 2 were related to a decreased risk of DKD. Additionally, DKD has a discernible effect on the abundance of 14 GM taxa. Most of these associations are in accordance with the findings of observational studies; however, the directions of the effects were not distinguished from each other previously. For example, Wang et al. [24] reported abnormal relative abundances of *Akkermansia* and Lachnospiraceae in patients with DKD. In our study, *Akkermansia* might have contributed to the development of DKD, but the abnormal abundance of Lachnospiraceae NC2004 and Lachnospiraceae UCG010 might be a consequence of DKD. In addition, we found that the abundance of the phylum Proteobacteria could be affected by DKD. The marked increase in the abundance of this phylum in patients with DKD compared to the non-DKD group reported by He et al. [25] might thus be a result, not a cause, of DKD.

*Akkermansia*, a member of the phylum Verrucomicrobia, showed an adverse link with DKD development in our study. Several studies have suggested detrimental effects of *Akkermansia*. A meta-analysis including 578 patients with DKD and 444 controls from 16 studies found enrichment of *Akkermansia* in DKD [24]. Additionally, *Akkermansia* abundance was elevated in DKD mice, and this correlated with renal damage indicators and could be mitigated with herbal medicines used for treating DKD [26, 27]. However, *Akkermansia*, particularly *Akkermansia muciniphila*, has been recognised as a probiotic and negatively related to Type 2 diabetes, obesity, and hypertension in other studies [28–31]. Supplementation with *A. muciniphila* in humans or mice may improve metabolic parameters and reverse diet–induced disorders [32, 33]. These findings highlight the complexity of the role of *Akkermansia* and warrant further investigation.

In our MR analysis, we observed a causal association between Erysipelotrichaceae UCG003 and both DKD and GFR. Similarly, Ruminococcaceae UCG014 was found to be simultaneously associated with DKD and UACR. Both taxa have been implicated in renal injury as per prior studies. Erysipelotrichaceae UCG003 has been related to chronic kidney disease [34], while a study by Vaziri et al. [35] has highlighted an association between Ruminococcaceae and estimated GFR in a population-based cohort. Meanwhile, both Erysipelotrichaceae and Ruminococcaceae are known butyrate-producing bacteria that belong to the phylum Firmicutes [36, 37]. Butyrate serves as a preferred energy substrate for colonic epithelial cells and is crucial for the maintenance of intestinal barrier integrity. Experimental rodent models have shown that butyrate can inhibit NF-*κ*B activation, ameliorate hyperglycaemia and insulin resistance, and protect against renal damage [38]. Furthermore, the restoration of butyrate levels has also been shown to be beneficial for hypertension. As reported by Wang et al. [39], sodium butyrate has the potential to mitigate hypertension induced by angiotensin II and improves the associated renal injury, encompassing urinary albumin excretion, glomerulosclerosis, and renal fibrosis.

Interestingly, when we separately analysed renal complications in the two main types of diabetes, we found that most causal relationships identified between GM and DKD were valid in Type 2 diabetes with renal complications, but not in Type 1 diabetes with renal complications. Given the higher prevalence of Type 2 diabetes, this disparity may be attributed to the predominance of Type 2 diabetics in the studied cohort for DKD. Although the GM of patients with renal complications and different types of diabetes has not been reported, differences in GM structure and composition between the two main types of diabetes have been demonstrated in our previous study [40] and several other studies [41–43]. Notably, adult-onset Type 1 diabetes was characterised by a significant reduction in SCFA-producing bacteria, which is linked to islet autoimmunity and pancreatic beta-cell dysfunction [44].

Our study had several limitations that may have affected the depth of our analysis. First, we employed a relatively flexible IV screening criterion (*p* < 1 × 10^−5^), which may have reduced the statistical power of the MR analysis. To mitigate potential false positives, we applied FDR correction. Second, although the majority of the participants in this study were Europeans, a small percentage (< 17.7%) of the participants of different races was included in the MiBioGen project, which might have resulted in a population stratification bias. However, we faced constraints as large GM GWAS data for the same racial groups are unavailable. Third, our MR analysis was based on summary-level data rather than individual-level data. Lastly, we could only obtain data at the genus level from the MiBioGen project, which lacks species- or strain-level information.

Collectively, our findings in this study provide further evidence for the bidirectional causality between GM and DKD, using an MR approach for the first time. We identified not only specific bacterial taxa that might contribute to the pathogenesis of DKD but also other microbial taxa, the abundance of which might be altered by DKD. Our results offer novel clues for potential mechanistic research and treatment targets for DN, with a future focus on the GM.

## Data Availability

We used summary-level GWAS data from public databases for the MR analyses in our study: MiBioGen repository (https://mibiogen.gcc.rug.nl/) (accessed on September 15, 2023), IEU Open GWAS project (https://gwas.mrcieu.ac.uk/) (accessed on September 17, 2023), and CKDGen consortium (http://ckdgen.imbi.uni-freiburg.de/) (accessed on October 7, 2023).

## Supporting Information

Supporting Information 1 Figure S1: scatter plots of forward MR analysis showing the effect of gut microbiota on diabetic kidney disease.

Supporting Information 2 Figure S2: scatter plots of reverse MR analysis showing the effect of diabetic kidney disease on gut microbiota.

Supporting Information 3 Figure S3: leave-one-out stability tests' causal estimates of exposure (gut microbiota) on outcomes (diabetic kidney disease).

Supporting Information 4 Figure S4: leave-one-out stability tests' causal estimates of exposure (diabetic kidney disease) on outcomes (gut microbiota).

Supporting Information 5 Table S1: STROBE-MR checklist of the present study. Table S2: detailed information of genome-wide association studies included in this study. Table S3: information about the selected SNPs of the forward MR analysis. Table S4: forward MR results of the causal effect of gut microbiota on diabetic kidney disease. Table S5: forward MR results of the causal effect of gut microbiota on four traits associated with diabetic kidney disease. Table S6: information about the selected SNPs of the reverse MR analysis. Table S7: reverse MR results of the causal effects of diabetic kidney disease on gut microbiota.
