# Supplementary figures and images for: Genetic Evidence for the Causal Relationship Between Gut Microbiota and Diabetic Kidney Disease: A Bidirectional, Two-Sample Mendelian Randomisation Study
Source: J Diabetes Res. 2024 Oct 23;2024:4545595. doi: 10.1155/2024/4545595 (PMC11524706; doi:10.1155/2024/4545595)

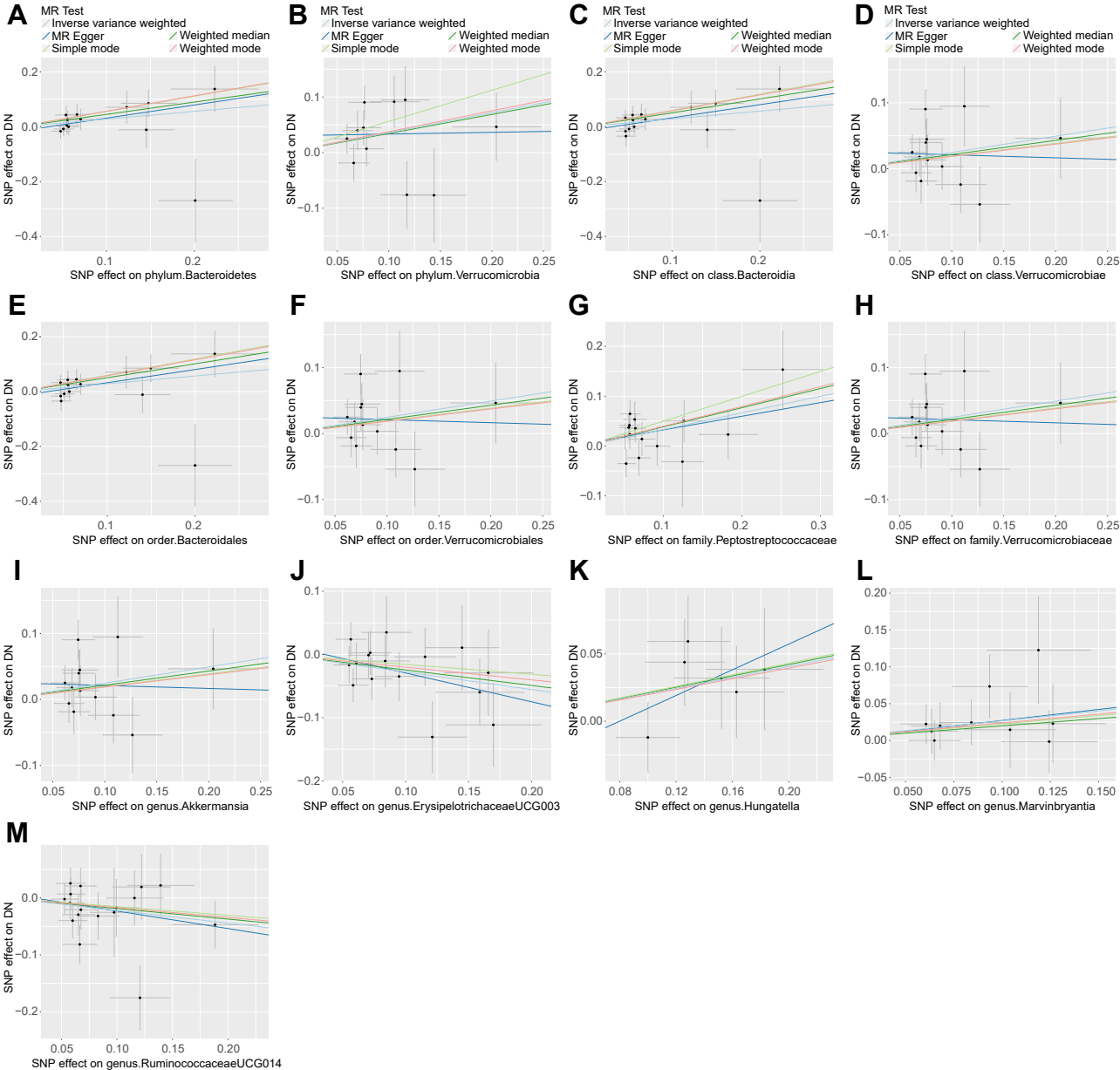

Supplement: Supporting Information 1 — Figure S1: scatter plots of forward MR analysis showing the effect of gut microbiota on diabetic kidney disease. [file 4545595.f1.pdf]

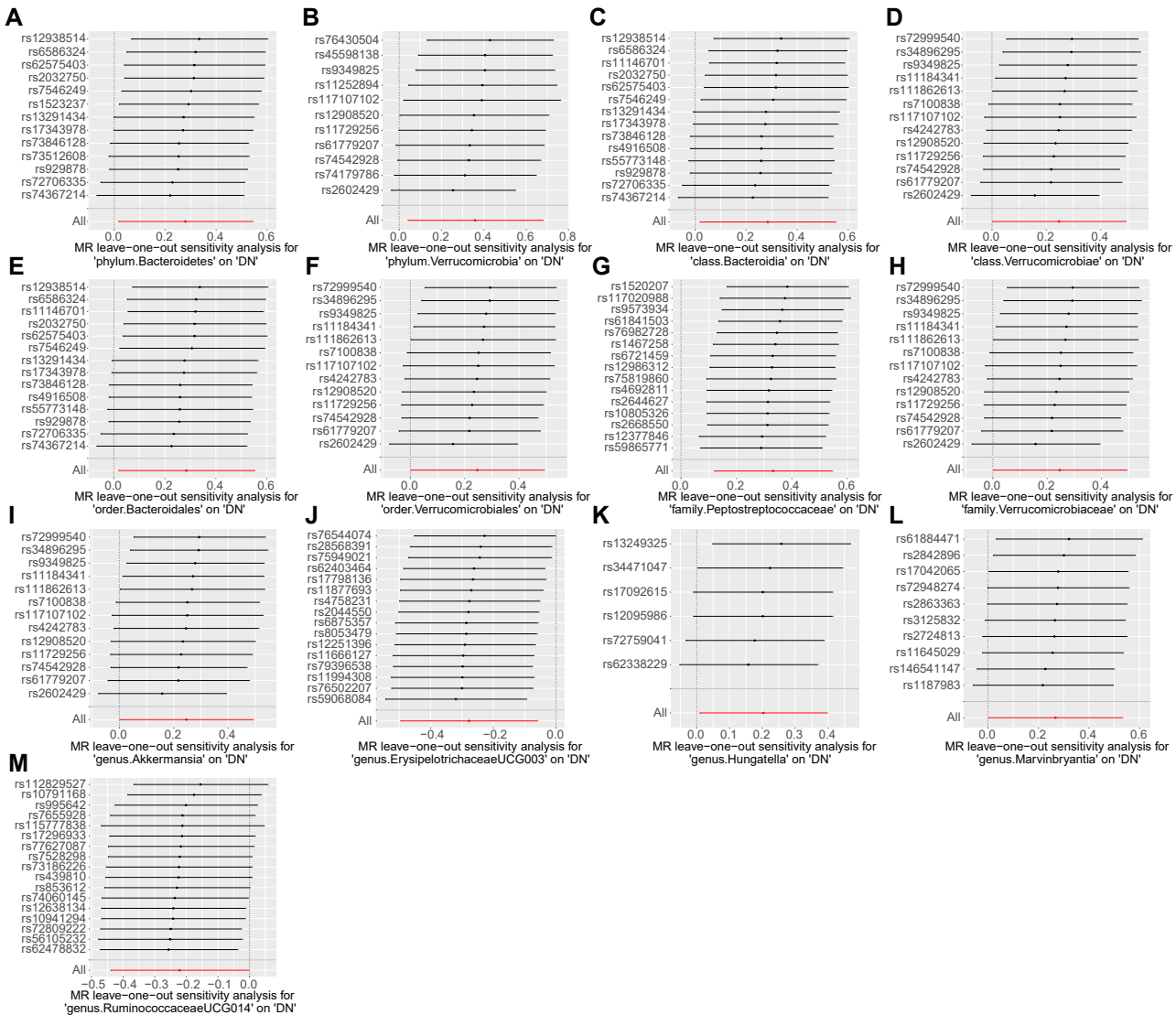

Supplement: Supporting Information 2 — Figure S2: scatter plots of reverse MR analysis showing the effect of diabetic kidney disease on gut microbiota. [file 4545595.f2.pdf]

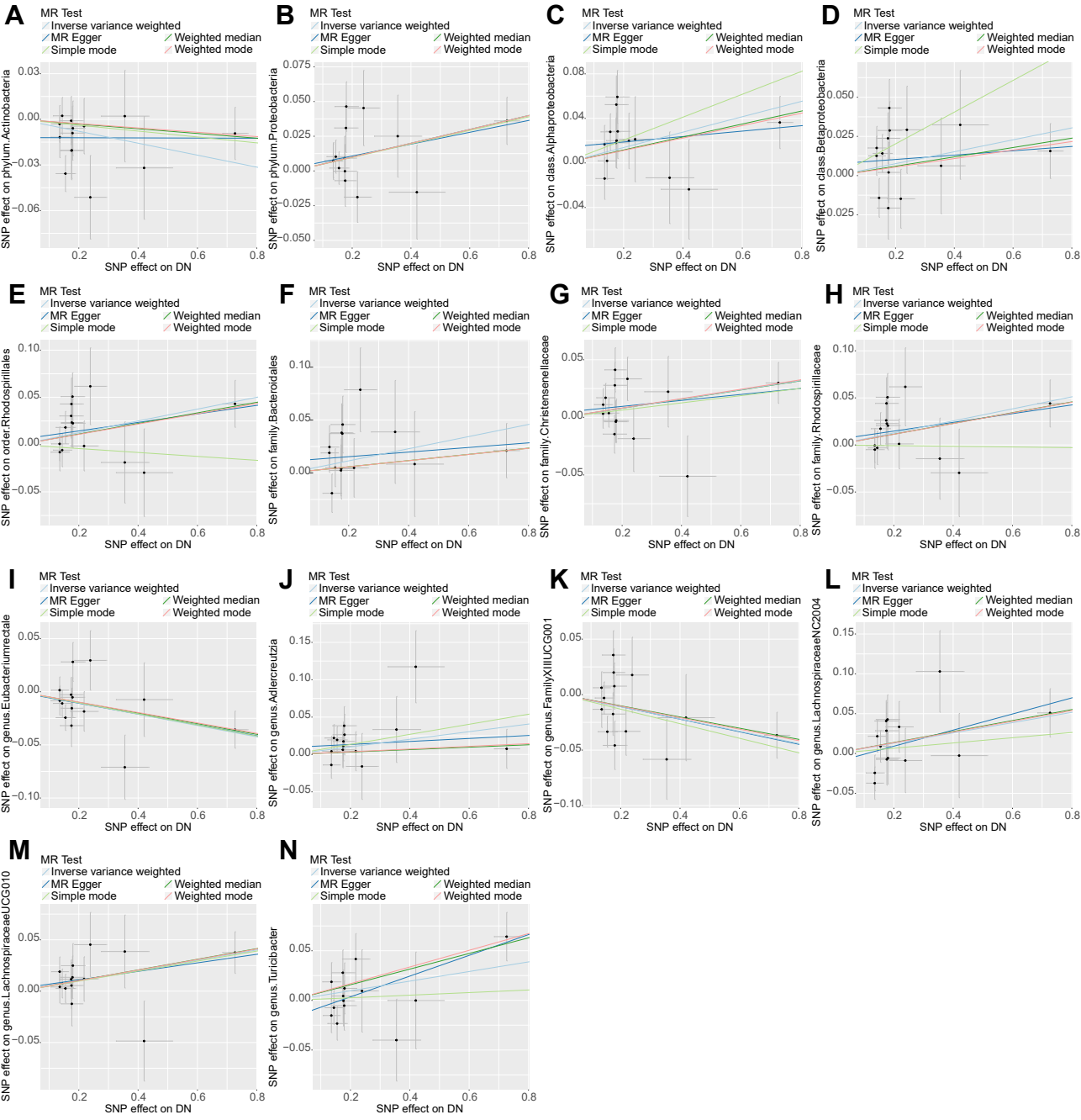

Supplement: Supporting Information 3 — Figure S3: leave-one-out stability tests' causal estimates of exposure (gut microbiota) on outcomes (diabetic kidney disease). [file 4545595.f3.pdf]

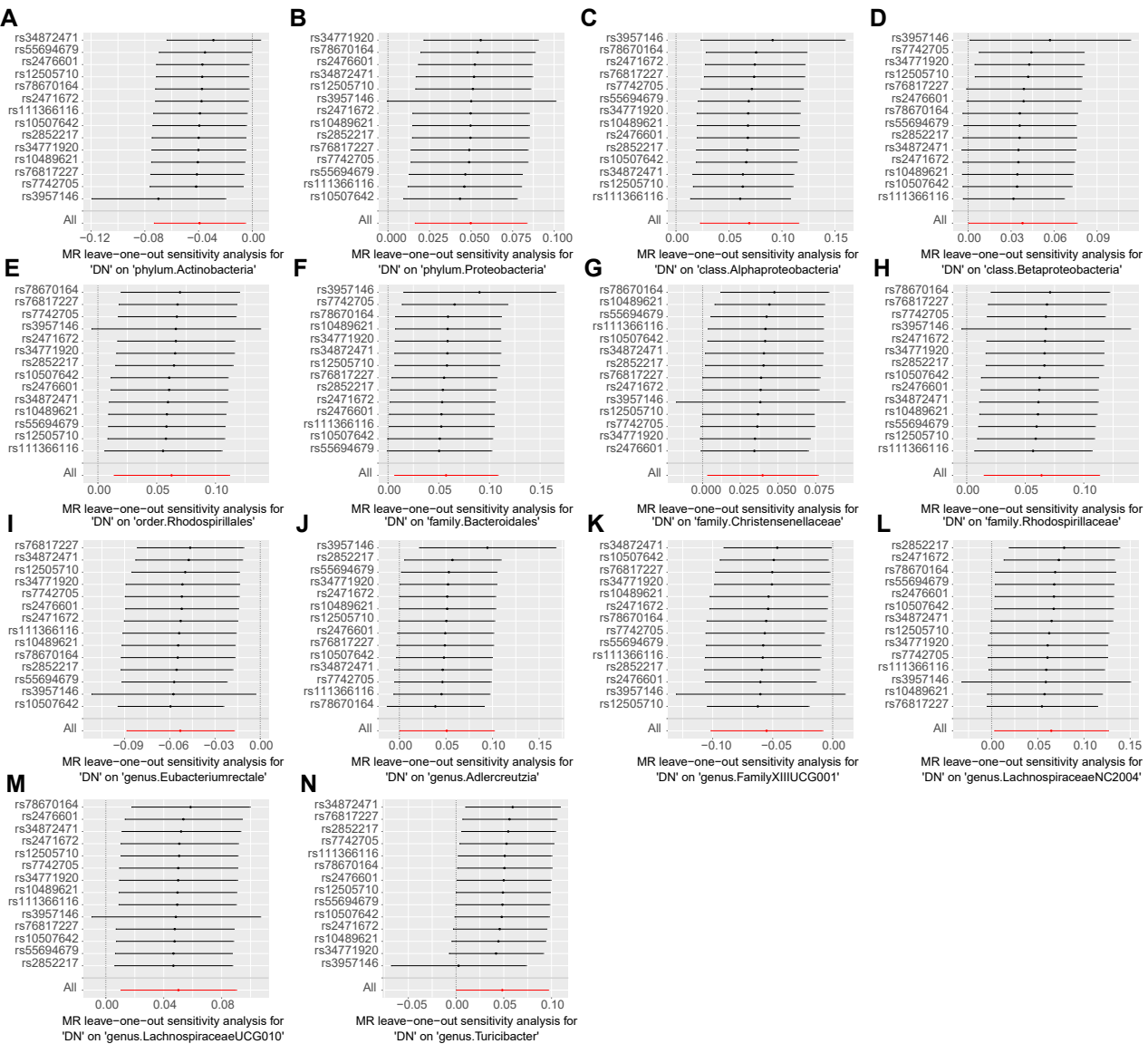

Supplement: Supporting Information 4 — Figure S4: leave-one-out stability tests' causal estimates of exposure (diabetic kidney disease) on outcomes (gut microbiota). [file 4545595.f4.pdf]
