# Supplementary material for: Genetic Evidence for the Causal Relationship Between Gut Microbiota and Diabetic Kidney Disease: A Bidirectional, Two-Sample Mendelian Randomisation Study
Source: J Diabetes Res. 2024 Oct 23;2024:4545595. doi: 10.1155/2024/4545595 (PMC11524706; doi:10.1155/2024/4545595)
Supplement: Supporting Information 5 — Table S1: STROBE-MR checklist of the present study. Table S2: detailed information of genome-wide association studies included in this study. Table S3: information about the selected SNPs of the forward MR analysis. Table S4: forward MR results of the causal effect of gut microbiota on diabetic kidney disease. Table S5: forward MR results of the causal effect of gut microbiota on four traits associated with diabetic kidney disease. Table S6: information about the selected SNPs of the reverse MR analysis. Table S7: reverse MR results of the causal effects of diabetic kidney disease on gut microbiota. [file 4545595.f5.docx]

**Supplementary Tables**

Table S1: STROBE-MR checklist of the present study

Table S2: Detailed information of genome-wide association studies included in this study

Table S3: Information about the selected SNPs of the forward MR analysis

Table S4: Forward MR results of the causal effect of gut microbiota on diabetic kidney disease

Table S5: Forward MR results of the causal effect of gut microbiota on four traits associated with diabetic kidney disease

Table S6: Information about the selected SNPs of the reverse MR analysis

Table S7: Reverse MR results of the causal effects of diabetic kidney disease on gut microbiota.

| **Table S1. STROBE-MR checklist of the present study.** | | | |  | |
| --- | --- | --- | --- | --- | --- |
| **No.** | **Section** | **Checklist item** | **Position** | |  |
| 1 | Title and abstract | Indicate MR as the study’s design in the title and the abstract as a main purpose of the study. | Title, Abstract | |  |
| 2 | Background | Explain the scientific background and rationale for the reported study. Explain the exposure and a plausible potential causal relationship between exposure and outcome. Justify why MR is a helpful method to address the study question. | Introduction:P1-P2 | |  |
| 3 | Objectives | State specific objectives clearly, including prespecified causal hypotheses. State that MR is a method that intends to estimate causal effects. | Introduction: P2 | |  |
| 4a | Study design and data sourses | Setting: Describe the study design (two-sample MR) and the underlying population. Describe the setting, locations, and relevant dates, including periods of recruitment, exposure, follow-up, and data collection. | Materials and methods: *Study design, Data sources*, Figure 1 | |  |
| 4b |  | Participants: Report the eligibility criteria and the sources and methods of selection of participants. Report the sample size and whether any power or sample size calculations were carried out prior to the main analysis. | Materials and methods: *Study design, Data sources, Ethical approval* | |  |
| 4c |  | Describe measurement, quality control, and selection of genetic variants. | Materials and methods: *Selection of eligible instrumental variables* | |  |
| 4d |  | For each exposure, outcome, and other relevant variables, describe methods of assessment and diagnostic criteria for diseases. | Materials and methods: *Data sources* | |  |
| 4e |  | Provide details of ethics committee approval and participant informed consent, if relevant. | Materials and methods: *Ethical approval* | |  |
| 5 | Assumptions | Explicitly state the 3 core instrumental variable (IV) assumptions for the main analysis (relevance, independence, and exclusion restriction), as well assumptions for any additional or sensitivity analysis. | Materials and methods: *Selection of eligible instrumental variables, Statistical methods and sensitivity analysis* | |  |
| 6a | Statistical methods: | Describe how quantitative variables were handled in the analyses. | Materials and methods: *Selection of eligible instrumental variable* | |  |
| 6b |  | Describe how genetic variants were handled in the analyses and, if applicable, how their weights were selected |  |  |  |
| 6c |  | Describe the MR estimator and related statistics. Detail the included covariates and, in case of 2-sample MR, whether the same covariate set was used for adjustment in the 2 samples. | Materials and methods: *Statistical methods and sensitivity analysis* | |  |
| 6d |  | Explain how missing data were addressed. |  |  |  |
| 6e |  | Indicate how multiple testing was addressed (Bonferroni correction) |  |  |  |
| 7 | Assessment of assumptions | Describe any methods or prior knowledge used to assess the assumptions or justify their validity |  |  |  |
| 8 | Sensitivity analyses and additional analyses | Describe any sensitivity analyses or additional analyses performed (eg, comparison of effect estimates from different approaches, independent replication, bias analytic techniques, validation of instruments, simulations). |  |  |  |
| 9a | Software and preregistration | Name statistical software and package(s), including version and settings used. |  |  |  |
| 9b |  | State whether the study protocol and details were preregistered (as well as when and where). | N/A | |  |
| 10a | Descriptive data | Report the numbers of individuals at each stage of included studies and reasons for exclusion. Use of a flow diagram. | Figure 1 | |  |
| 10b |  | Report summary statistics for phenotypic exposure , outcomes, and other relevant variables (eg, means, SDs, proportions). | Materials and methods: *Study design, Data sources*, Figure 1 | |  |
| 10c |  | If the data sources include meta-analyses of previous studies, provide the assessments of heterogeneity across these studies. | N/A | |  |
| 10d |  | For 2-sample MR:  i. Provide justification of the similarity of the genetic variant–exposure associations between the exposure and outcome samples.  ii. Provide information on the number of individuals who overlap between the exposure and outcome studies. | Materials and methods | |  |
| 11a | Main results | Report the associations between genetic variant and exposure and between genetic variant and outcome, preferably on an interpretable scale. | Results, Table S2 | |  |
| 11b |  | Report MR estimates of the relationship between exposure and outcome and the measures of uncertainty from the MR analysis, on an interpretable scale, such as odds ratio or relative risk per SD difference. | Results-Figure 2-3, Table S3, Table S5 | |  |
| 11c |  | If relevant, consider translating estimates of relative risk into absolute risk for a meaningful time period. | N/A | |  |
| 11d |  | Consider plots to visualize results (eg, forest plot, scatterplot of associations between genetic variants and outcome vs between genetic variants and exposure). | Figure 2-3, Figure S1-4 | |  |
| 12a | Assessment of assumptions | Report the assessment of the validity of the assumptions by removing confounders-related SNPs. | Results: *Sensitivity analysis* | |  |
| 12b |  | Report any additional statistics (eg, assessments of heterogeneity across genetic variants, such as I^2^, Q statistic). | Results: *Sensitivity analysis* | |  |
| 13a | Sensitivity analyses and additional analyses | Report any sensitivity analyses to assess the robustness of the main results to violations of the assumptions. | Results: *Sensitivity analysis* | |  |
| 13b |  | Report results from other sensitivity analyses or additional analyses | Results: *Sensitivity analysis* | |  |
| 13c |  | Report any assessment of the direction of the causal relationship. | Figure 2-3 | |  |
| 13d |  | When relevant, report and compare with estimates from other RCTs and meta-analyses. | N/A | |  |
| 13e |  | Consider additional plots to visualize results. | All Figure and Table | |  |
| 14 | Key results | Summarize key results with reference to study objectives. | Discussion: Paragraph 2-4 | |  |
| 15 | Limitations | Discuss limitations of the study, taking into account the validity of the IV assumptions, other sources of potential bias, and imprecision. Discuss both direction and magnitude of any potential bias and any efforts to address them. | Discussion: Paragraph 5 | |  |
| 16a | Interpretation | Meaning: Give a cautious overall interpretation of results in the context of their limitations and in comparison with other studies. | Discussion: Paragraph 1-2 | |  |
| 16b |  | Mechanism: Discuss underlying biological mechanisms that could drive a potential causal relationship between the investigated exposure and the outcome, and whether the gene-environment equivalence assumption is reasonable. Use causal language carefully, clarifying that IV estimates may provide causal effects only under certain assumptions. | Discussion | |  |
| 16c |  | Clinical relevance: Discuss whether the results have clinical or public policy relevance, and to what extent they inform effect sizes of possible interventions. |  |  |  |
| 17 |  | Discuss the generalizability of the study results (a) to other populations, (b) across other exposure periods/timings, and (c) across other levels of exposure. |  |  |  |
| 18 | Funding | Describe sources of funding and the role of funders in the present study. | Acknowledgment: *Funding* | |  |
| 19 | Data and data sharing | Provide the data used to perform all analyses or report where and how the data can be accessed, and reference these sources in the article. | Materials and methods: *Data sources* | |  |
| 20 | Conflicts of interest | All authors should declare all potential conflicts of interest. | Conflict of Interest | |  |
|  |  |  |  | |  |
| **Three fundamental ssumptions** | |  |  | |  |
| a. The instrument variables (IVs) must be associated with exposures; | | | |  | |
| b. The IVs must not be associated with any confounders, such as body mass index (BMI), smoking, and hypertension; | | | | | |
| c. The IVs must influence the outcomes only through exposures and not through any direct or alternative pathways. | | | | | |

| **Table S2: Detailed information of genome-wide association studies included in this study.** | | | | | | | |  |  |  |  |
| --- | --- | --- | --- | --- | --- | --- | --- | --- | --- | --- | --- |
|  |  |  |  |  |  |  |  |  |  |  |  |
| **Year** | **Trait** | **Population** | **Sample size** | **Numbers of SNPs** | **Web source** | **PMID** | **GWAS ID** |  |  |  |  |
| 2021 | Gut microbiota | European, Hispanic, Middle Eastern,Asian and African | 18,340 |  | https://mibiogen.gcc.rug.nl/ | 33462485 |  |  |  |  |  |
| 2021 | Diabetic kidney disease | European | 184,987 | 16,380,336 | https://gwas.mrcieu.ac.uk/datasets/ |  | [finn-b-DM_NEPHROPATHY_EXMORE](https://gwas.mrcieu.ac.uk/datasets/finn-b-DM_NEPHROPATHY_EXMORE/) |  |  |  |  |
| 2021 | Type 1 diabetes with renal complications | Europeancc | 184,148 | 16,380,334 | https://gwas.mrcieu.ac.uk/datasets/ |  | finn-b-E4_DM1REN |  |  |  |  |
| 2021 | Type 2 diabetes with renal complicationsc | European | 184,481 | 16,380,337 | https://gwas.mrcieu.ac.uk/datasets/ |  | finn-b-E4_DM2REN |  |  |  |  |
| 2016 | Glomerular filtration rate in diabetics | European | 11,522 | 2,169,300 | https://gwas.mrcieu.ac.uk/datasets/ | 26831199 | ebi-a-GCST003373 |  |  |  |  |
| 2015 | Urinary albumin-to-creatinine ratio in diabetes | European | 5,825 |  | http://ckdgen.imbi.uni-freiburg.de/ | 26631737 |  |  |  |  |  |

| **Table S3 Information about the selected SNPs of the forward MR analysis.** | | | | | | | | | | | | | | |
| --- | --- | --- | --- | --- | --- | --- | --- | --- | --- | --- | --- | --- | --- | --- |
|  |  |  |  |  |  |  |  |  |  |  |  |  |  |  |
| **cccccccccExposure** | **Outcome** | **SNP** | **Chr** | **Position** | **Effect allele** | **Other allele** | **MAF** | **Beta.exposure** | **Beta.outcome** | **SE.exposure** | **SE.outcome** | **P-value.exposure** | **P-value.outcome** | **mr_keep** |
| Phylum Bacteroidetes | Diabetic kidney disease | rs12938514 | 17 | 6460281 | A | G | 0.00755 | -0.201088983 | 0.2691 | 0.041599463 | 0.15 | 1.33862E-06 | 0.0727595 | TRUE |
| Phylum Bacteroidetes | Diabetic kidney disease | rs13291434 | 9 | 116888634 | T | C | 0.1424 | 0.070581793 | 0.027 | 0.014978049 | 0.0372 | 2.44878E-06 | 0.4686 | TRUE |
| Phylum Bacteroidetes | Diabetic kidney disease | rs1523237 | 15 | 26381111 | G | T | 0.2495 | -0.054910561 | -0.005 | 0.012295811 | 0.0299 | 7.97723E-06 | 0.8671 | TRUE |
| Phylum Bacteroidetes | Diabetic kidney disease | rs17343978 | 22 | 27433885 | A | C | 0.204 | -0.055604102 | -0.0247 | 0.012027223 | 0.032 | 3.77889E-06 | 0.44 | TRUE |
| Phylum Bacteroidetes | Diabetic kidney disease | rs2032750 | 2 | 53831026 | T | C | 0.4615 | -0.051085659 | 0.0083 | 0.010680423 | 0.026 | 1.72602E-06 | 0.7508 | TRUE |
| Phylum Bacteroidetes | Diabetic kidney disease | rs62575403 | 9 | 136493820 | C | T | 0.04231 | 0.145423872 | -0.0113 | 0.031107867 | 0.0653 | 2.94203E-06 | 0.8632 | TRUE |
| Phylum Bacteroidetes | Diabetic kidney disease | rs6586324 | 21 | 43942135 | T | C | 0.4262 | 0.047779239 | -0.0164 | 0.010543692 | 0.0261 | 5.85533E-06 | 0.5285 | TRUE |
| Phylum Bacteroidetes | Diabetic kidney disease | rs72706335 | 1 | 157495438 | T | C | 0.02558 | -0.223193178 | -0.1374 | 0.049338227 | 0.0826 | 6.07571E-06 | 0.0963208 | TRUE |
| Phylum Bacteroidetes | Diabetic kidney disease | rs73512608 | 13 | 70522683 | G | A | 0.05305 | -0.123117207 | -0.0708 | 0.02367433 | 0.0575 | 1.98805E-07 | 0.2179 | TRUE |
| Phylum Bacteroidetes | Diabetic kidney disease | rs73846128 | 3 | 89340254 | A | G | 0.1489 | -0.066426181 | -0.0445 | 0.013348134 | 0.0363 | 6.47646E-07 | 0.2208 | TRUE |
| Phylum Bacteroidetes | Diabetic kidney disease | rs74367214 | 7 | 105897170 | T | A | 0.07821 | -0.147871528 | -0.0852 | 0.031155717 | 0.0484 | 2.07265E-06 | 0.0782799 | TRUE |
| Phylum Bacteroidetes | Diabetic kidney disease | rs7546249 | 1 | 176792690 | T | A | 0.2936 | -0.056917331 | 0.0002 | 0.01183824 | 0.0284 | 1.52508E-06 | 0.9957 | TRUE |
| Phylum Bacteroidetes | Diabetic kidney disease | rs929878 | 16 | 74290641 | C | T | 0.2133 | -0.054000099 | -0.0426 | 0.012152588 | 0.0315 | 8.85047E-06 | 0.1762 | TRUE |
| Phylum Verrucomicrobia | Diabetic kidney disease | rs11252894 | 10 | 5073050 | A | C | 0.2411 | 0.078405494 | 0.0069 | 0.016276917 | 0.0303 | 1.45751E-06 | 0.8197 | TRUE |
| Phylum Verrucomicrobia | Diabetic kidney disease | rs117107102 | 18 | 49473635 | A | G | 0.04764 | 0.204282775 | 0.0464 | 0.042756505 | 0.0604 | 1.77208E-06 | 0.442 | TRUE |
| Phylum Verrucomicrobia | Diabetic kidney disease | rs11729256 | 4 | 95027272 | T | C | 0.1696 | 0.069698414 | 0.0397 | 0.01469534 | 0.0344 | 2.10688E-06 | 0.2488 | TRUE |
| Phylum Verrucomicrobia | Diabetic kidney disease | rs12908520 | 15 | 97570657 | G | A | 0.4268 | 0.059491573 | 0.025 | 0.012823012 | 0.0261 | 3.49357E-06 | 0.3382 | TRUE |
| Phylum Verrucomicrobia | Diabetic kidney disease | rs2602429 | 16 | 81063149 | C | T | 0.2612 | 0.076414823 | 0.0904 | 0.015348672 | 0.0295 | 6.40475E-07 | 0.00216701 | TRUE |
| Phylum Verrucomicrobia | Diabetic kidney disease | rs45598138 | 1 | 55522083 | C | A | 0.02536 | -0.143901454 | 0.0768 | 0.030540001 | 0.0836 | 2.45417E-06 | 0.3589 | TRUE |
| Phylum Verrucomicrobia | Diabetic kidney disease | rs61779207 | 1 | 41074472 | G | A | 0.2236 | -0.075525262 | -0.0447 | 0.016438461 | 0.0311 | 4.33946E-06 | 0.1508 | TRUE |
| Phylum Verrucomicrobia | Diabetic kidney disease | rs74179786 | 20 | 15746378 | G | A | 0.09028 | -0.105283337 | -0.0915 | 0.023767493 | 0.0463 | 9.43555E-06 | 0.0482903 | TRUE |
| Phylum Verrucomicrobia | Diabetic kidney disease | rs74542928 | 4 | 100544188 | T | C | 0.04797 | 0.115994333 | 0.0947 | 0.023132372 | 0.0605 | 5.32068E-07 | 0.1178 | TRUE |
| Phylum Verrucomicrobia | Diabetic kidney disease | rs76430504 | 5 | 40438158 | T | C | 0.0484 | -0.117530089 | 0.076 | 0.025458042 | 0.0599 | 3.90042E-06 | 0.2047 | TRUE |
| Phylum Verrucomicrobia | Diabetic kidney disease | rs9349825 | 6 | 56341481 | A | G | 0.1895 | -0.066024311 | 0.0187 | 0.014391139 | 0.0331 | 4.47846E-06 | 0.5727 | TRUE |
| Class Bacteroidia | Diabetic kidney disease | rs11146701 | 10 | 39062269 | A | G | 0.3426 | 0.047359582 | -0.0345 | 0.010540996 | 0.0342 | 7.02616E-06 | 0.3129 | TRUE |
| Class Bacteroidia | Diabetic kidney disease | rs12938514 | 17 | 6460281 | A | G | 0.00755 | -0.199979189 | 0.2691 | 0.041602386 | 0.15 | 1.53276E-06 | 0.0727595 | TRUE |
| Class Bacteroidia | Diabetic kidney disease | rs13291434 | 9 | 116888634 | T | C | 0.1424 | 0.069465573 | 0.027 | 0.014978949 | 0.0372 | 3.52569E-06 | 0.4686 | TRUE |
| Class Bacteroidia | Diabetic kidney disease | rs17343978 | 22 | 27433885 | A | C | 0.204 | -0.055205684 | -0.0247 | 0.012027767 | 0.032 | 4.43558E-06 | 0.44 | TRUE |
| Class Bacteroidia | Diabetic kidney disease | rs2032750 | 2 | 53831026 | T | C | 0.4615 | -0.050839276 | 0.0083 | 0.010680731 | 0.026 | 1.93683E-06 | 0.7508 | TRUE |
| Class Bacteroidia | Diabetic kidney disease | rs4916508 | 3 | 195936789 | G | A | 0.4239 | -0.046672941 | -0.0326 | 0.010531247 | 0.0261 | 9.34266E-06 | 0.2113 | TRUE |
| Class Bacteroidia | Diabetic kidney disease | rs55773148 | 13 | 70523029 | G | A | 0.05305 | -0.121513597 | -0.0708 | 0.023675903 | 0.0575 | 2.8611E-07 | 0.2179 | TRUE |
| Class Bacteroidia | Diabetic kidney disease | rs62575403 | 9 | 136493820 | C | T | 0.04231 | 0.140054866 | -0.0113 | 0.031112824 | 0.0653 | 6.74706E-06 | 0.8632 | TRUE |
| Class Bacteroidia | Diabetic kidney disease | rs6586324 | 21 | 43942135 | T | C | 0.4262 | 0.046825104 | -0.0164 | 0.010544205 | 0.0261 | 8.96093E-06 | 0.5285 | TRUE |
| Class Bacteroidia | Diabetic kidney disease | rs72706335 | 1 | 157495438 | T | C | 0.02558 | -0.222409754 | -0.1374 | 0.049345816 | 0.0826 | 6.56994E-06 | 0.0963208 | TRUE |
| Class Bacteroidia | Diabetic kidney disease | rs73846128 | 3 | 89340254 | A | G | 0.1489 | -0.064852276 | -0.0445 | 0.013349001 | 0.0363 | 1.18451E-06 | 0.2208 | TRUE |
| Class Bacteroidia | Diabetic kidney disease | rs74367214 | 7 | 105897170 | T | A | 0.07821 | -0.149447961 | -0.0852 | 0.031153242 | 0.0484 | 1.60908E-06 | 0.0782799 | TRUE |
| Class Bacteroidia | Diabetic kidney disease | rs7546249 | 1 | 176792690 | T | A | 0.2936 | -0.056711256 | 0.0002 | 0.01183859 | 0.0284 | 1.66472E-06 | 0.9957 | TRUE |
| Class Bacteroidia | Diabetic kidney disease | rs929878 | 16 | 74290641 | C | T | 0.2133 | -0.054853206 | -0.0426 | 0.012152954 | 0.0315 | 6.37455E-06 | 0.1762 | TRUE |
| Class Verrucomicrobiae | Diabetic kidney disease | rs11184341 | 1 | 105422565 | G | C | 0.2743 | 0.065534193 | -0.0061 | 0.014223078 | 0.0289 | 4.07351E-06 | 0.8327 | TRUE |
| Class Verrucomicrobiae | Diabetic kidney disease | rs111862613 | 12 | 130309670 | T | C | 0.1776 | 0.090698957 | 0.0034 | 0.019674596 | 0.0346 | 4.02761E-06 | 0.9215 | TRUE |
| Class Verrucomicrobiae | Diabetic kidney disease | rs117107102 | 18 | 49473635 | A | G | 0.04764 | 0.20468288 | 0.0464 | 0.043157251 | 0.0604 | 2.10865E-06 | 0.442 | TRUE |
| Class Verrucomicrobiae | Diabetic kidney disease | rs11729256 | 4 | 95027272 | T | C | 0.1696 | 0.074979802 | 0.0397 | 0.015017677 | 0.0344 | 5.95195E-07 | 0.2488 | TRUE |
| Class Verrucomicrobiae | Diabetic kidney disease | rs12908520 | 15 | 97570657 | G | A | 0.4268 | 0.061892558 | 0.025 | 0.013094626 | 0.0261 | 2.28353E-06 | 0.3382 | TRUE |
| Class Verrucomicrobiae | Diabetic kidney disease | rs2602429 | 16 | 81063149 | C | T | 0.2612 | 0.074684437 | 0.0904 | 0.015619438 | 0.0295 | 1.73987E-06 | 0.00216701 | TRUE |
| Class Verrucomicrobiae | Diabetic kidney disease | rs34896295 | 9 | 139060991 | A | T | 0.1079 | -0.108532331 | 0.024 | 0.024341603 | 0.0417 | 8.24515E-06 | 0.5645 | TRUE |
| Class Verrucomicrobiae | Diabetic kidney disease | rs4242783 | 10 | 5064327 | G | A | 0.2833 | 0.068929219 | 0.0178 | 0.014769297 | 0.0288 | 3.05538E-06 | 0.5369 | TRUE |
| Class Verrucomicrobiae | Diabetic kidney disease | rs61779207 | 1 | 41074472 | G | A | 0.2236 | -0.075830853 | -0.0447 | 0.016776157 | 0.0311 | 6.1794E-06 | 0.1508 | TRUE |
| Class Verrucomicrobiae | Diabetic kidney disease | rs7100838 | 10 | 60876067 | G | A | 0.1359 | 0.076768771 | 0.0134 | 0.017271386 | 0.0377 | 8.7952E-06 | 0.7228 | TRUE |
| Class Verrucomicrobiae | Diabetic kidney disease | rs72999540 | 2 | 240347720 | T | C | 0.05509 | -0.126896009 | 0.054 | 0.028408041 | 0.0566 | 7.93595E-06 | 0.34 | TRUE |
| Class Verrucomicrobiae | Diabetic kidney disease | rs74542928 | 4 | 100544188 | T | C | 0.04797 | 0.112163707 | 0.0947 | 0.023642195 | 0.0605 | 2.09314E-06 | 0.1178 | TRUE |
| Class Verrucomicrobiae | Diabetic kidney disease | rs9349825 | 6 | 56341481 | A | G | 0.1895 | -0.070403566 | 0.0187 | 0.014712789 | 0.0331 | 1.70821E-06 | 0.5727 | TRUE |
| Order Bacteroidales | Diabetic kidney disease | rs11146701 | 10 | 39062269 | A | G | 0.3426 | 0.047359582 | -0.0345 | 0.010540996 | 0.0342 | 0.00000703 | 0.3129 | TRUE |
| Order Bacteroidales | Diabetic kidney disease | rs12938514 | 17 | 6460281 | A | G | 0.00755 | -0.199979189 | 0.2691 | 0.041602386 | 0.15 | 0.00000153 | 0.0727595 | TRUE |
| Order Bacteroidales | Diabetic kidney disease | rs13291434 | 9 | 116888634 | T | C | 0.1424 | 0.069465573 | 0.027 | 0.014978949 | 0.0372 | 0.00000353 | 0.4686 | TRUE |
| Order Bacteroidales | Diabetic kidney disease | rs17343978 | 22 | 27433885 | A | C | 0.204 | -0.055205684 | -0.0247 | 0.012027767 | 0.032 | 0.00000444 | 0.44 | TRUE |
| Order Bacteroidales | Diabetic kidney disease | rs2032750 | 2 | 53831026 | T | C | 0.4615 | -0.050839276 | 0.0083 | 0.010680731 | 0.026 | 0.00000194 | 0.7508 | TRUE |
| Order Bacteroidales | Diabetic kidney disease | rs4916508 | 3 | 195936789 | G | A | 0.4239 | -0.046672941 | -0.0326 | 0.010531247 | 0.0261 | 0.00000934 | 0.2113 | TRUE |
| Order Bacteroidales | Diabetic kidney disease | rs55773148 | 13 | 70523029 | G | A | 0.05305 | -0.121513597 | -0.0708 | 0.023675903 | 0.0575 | 0.000000286 | 0.2179 | TRUE |
| Order Bacteroidales | Diabetic kidney disease | rs62575403 | 9 | 136493820 | C | T | 0.04231 | 0.140054866 | -0.0113 | 0.031112824 | 0.0653 | 0.00000675 | 0.8632 | TRUE |
| Order Bacteroidales | Diabetic kidney disease | rs6586324 | 21 | 43942135 | T | C | 0.4262 | 0.046825104 | -0.0164 | 0.010544205 | 0.0261 | 0.00000896 | 0.5285 | TRUE |
| Order Bacteroidales | Diabetic kidney disease | rs72706335 | 1 | 157495438 | T | C | 0.02558 | -0.222409754 | -0.1374 | 0.049345816 | 0.0826 | 0.00000657 | 0.0963208 | TRUE |
| Order Bacteroidales | Diabetic kidney disease | rs73846128 | 3 | 89340254 | A | G | 0.1489 | -0.064852276 | -0.0445 | 0.013349001 | 0.0363 | 0.00000118 | 0.2208 | TRUE |
| Order Bacteroidales | Diabetic kidney disease | rs74367214 | 7 | 105897170 | T | A | 0.07821 | -0.149447961 | -0.0852 | 0.031153242 | 0.0484 | 0.00000161 | 0.0782799 | TRUE |
| Order Bacteroidales | Diabetic kidney disease | rs7546249 | 1 | 176792690 | T | A | 0.2936 | -0.056711256 | 0.0002 | 0.01183859 | 0.0284 | 0.00000166 | 0.9957 | TRUE |
| Order Bacteroidales | Diabetic kidney disease | rs929878 | 16 | 74290641 | C | T | 0.2133 | -0.054853206 | -0.0426 | 0.012152954 | 0.0315 | 0.00000637 | 0.1762 | TRUE |
| Order Verrucomicrobiales | Diabetic kidney disease | rs11184341 | 1 | 105422565 | G | C | 0.2743 | 0.065534193 | -0.0061 | 0.014223078 | 0.0289 | 0.00000407 | 0.8327 | TRUE |
| Order Verrucomicrobiales | Diabetic kidney disease | rs111862613 | 12 | 130309670 | T | C | 0.1776 | 0.090698957 | 0.0034 | 0.019674596 | 0.0346 | 0.00000403 | 0.9215 | TRUE |
| Order Verrucomicrobiales | Diabetic kidney disease | rs117107102 | 18 | 49473635 | A | G | 0.04764 | 0.20468288 | 0.0464 | 0.043157251 | 0.0604 | 0.00000211 | 0.442 | TRUE |
| Order Verrucomicrobiales | Diabetic kidney disease | rs11729256 | 4 | 95027272 | T | C | 0.1696 | 0.074979802 | 0.0397 | 0.015017677 | 0.0344 | 0.000000595 | 0.2488 | TRUE |
| Order Verrucomicrobiales | Diabetic kidney disease | rs12908520 | 15 | 97570657 | G | A | 0.4268 | 0.061892558 | 0.025 | 0.013094626 | 0.0261 | 0.00000228 | 0.3382 | TRUE |
| Order Verrucomicrobiales | Diabetic kidney disease | rs2602429 | 16 | 81063149 | C | T | 0.2612 | 0.074684437 | 0.0904 | 0.015619438 | 0.0295 | 0.00000174 | 0.00216701 | TRUE |
| Order Verrucomicrobiales | Diabetic kidney disease | rs34896295 | 9 | 139060991 | A | T | 0.1079 | -0.108532331 | 0.024 | 0.024341603 | 0.0417 | 0.00000825 | 0.5645 | TRUE |
| Order Verrucomicrobiales | Diabetic kidney disease | rs4242783 | 10 | 5064327 | G | A | 0.2833 | 0.068929219 | 0.0178 | 0.014769297 | 0.0288 | 0.00000306 | 0.5369 | TRUE |
| Order Verrucomicrobiales | Diabetic kidney disease | rs61779207 | 1 | 41074472 | G | A | 0.2236 | -0.075830853 | -0.0447 | 0.016776157 | 0.0311 | 0.00000618 | 0.1508 | TRUE |
| Order Verrucomicrobiales | Diabetic kidney disease | rs7100838 | 10 | 60876067 | G | A | 0.1359 | 0.076768771 | 0.0134 | 0.017271386 | 0.0377 | 0.0000088 | 0.7228 | TRUE |
| Order Verrucomicrobiales | Diabetic kidney disease | rs72999540 | 2 | 240347720 | T | C | 0.05509 | -0.126896009 | 0.054 | 0.028408041 | 0.0566 | 0.00000794 | 0.34 | TRUE |
| Order Verrucomicrobiales | Diabetic kidney disease | rs74542928 | 4 | 100544188 | T | C | 0.04797 | 0.112163707 | 0.0947 | 0.023642195 | 0.0605 | 0.00000209 | 0.1178 | TRUE |
| Order Verrucomicrobiales | Diabetic kidney disease | rs9349825 | 6 | 56341481 | A | G | 0.1895 | -0.070403566 | 0.0187 | 0.014712789 | 0.0331 | 0.00000171 | 0.5727 | TRUE |
| Family Peptostreptococcaceae | Diabetic kidney disease | rs10805326 | 4 | 14324623 | G | A | 0.2943 | 0.0566637 | 0.0417 | 0.012275984 | 0.0284 | 0.00000392 | 0.1421 | TRUE |
| Family Peptostreptococcaceae | Diabetic kidney disease | rs117020988 | 7 | 46710977 | C | T | 0.08015 | 0.182411122 | 0.0231 | 0.037212625 | 0.0478 | 0.000000949 | 0.628599 | TRUE |
| Family Peptostreptococcaceae | Diabetic kidney disease | rs12377846 | 9 | 16786784 | C | A | 0.02871 | -0.252000426 | -0.1533 | 0.051162723 | 0.0771 | 0.000000842 | 0.0468198 | TRUE |
| Family Peptostreptococcaceae | Diabetic kidney disease | rs12986312 | 19 | 17414138 | T | G | 0.2993 | 0.057181848 | 0.0234 | 0.01259472 | 0.0282 | 0.00000562 | 0.4072 | TRUE |
| Family Peptostreptococcaceae | Diabetic kidney disease | rs1467258 | 17 | 38216487 | G | A | 0.188 | 0.072583976 | 0.0139 | 0.016232118 | 0.0332 | 0.00000776 | 0.676799 | TRUE |
| Family Peptostreptococcaceae | Diabetic kidney disease | rs1520207 | 3 | 151781175 | C | T | 0.4988 | 0.052565835 | -0.0353 | 0.011247608 | 0.0259 | 0.00000296 | 0.1736 | TRUE |
| Family Peptostreptococcaceae | Diabetic kidney disease | rs2644627 | 8 | 26726641 | C | G | 0.4284 | -0.055850562 | -0.0371 | 0.011224002 | 0.0261 | 0.000000649 | 0.1549 | TRUE |
| Family Peptostreptococcaceae | Diabetic kidney disease | rs2668550 | 4 | 54513106 | T | C | 0.1631 | -0.063454145 | -0.0533 | 0.014343022 | 0.0351 | 0.00000969 | 0.1287 | TRUE |
| Family Peptostreptococcaceae | Diabetic kidney disease | rs4692811 | 4 | 171180190 | C | T | 0.3402 | 0.064207491 | 0.0354 | 0.012691273 | 0.0273 | 0.000000421 | 0.1944 | TRUE |
| Family Peptostreptococcaceae | Diabetic kidney disease | rs59865771 | 16 | 89246670 | C | T | 0.3416 | -0.057376395 | -0.0645 | 0.012719207 | 0.0273 | 0.00000645 | 0.0180901 | TRUE |
| Family Peptostreptococcaceae | Diabetic kidney disease | rs61841503 | 10 | 17019559 | G | A | 0.1295 | 0.091965839 | -0.0002 | 0.016133103 | 0.0385 | 1.19E-08 | 0.9961 | TRUE |
| Family Peptostreptococcaceae | Diabetic kidney disease | rs6721459 | 2 | 67852836 | A | G | 0.5938 | 0.050982344 | 0.0187 | 0.011197512 | 0.0263 | 0.00000529 | 0.4768 | TRUE |
| Family Peptostreptococcaceae | Diabetic kidney disease | rs75819860 | 18 | 65324280 | T | A | 0.1143 | 0.126263781 | 0.0503 | 0.026838281 | 0.0415 | 0.00000254 | 0.226 | TRUE |
| Family Peptostreptococcaceae | Diabetic kidney disease | rs76982728 | 7 | 44312824 | T | C | 0.02128 | 0.124202832 | -0.0314 | 0.026692325 | 0.0901 | 0.00000327 | 0.727301 | TRUE |
| Family Peptostreptococcaceae | Diabetic kidney disease | rs9573934 | 13 | 77338074 | C | T | 0.165 | -0.068718903 | 0.0239 | 0.014153282 | 0.0348 | 0.0000012 | 0.4923 | TRUE |
| Family Verrucomicrobiaceae | Diabetic kidney disease | rs11184341 | 1 | 105422565 | G | C | 0.2743 | 0.065530075 | -0.0061 | 0.014223048 | 0.0289 | 0.00000408 | 0.8327 | TRUE |
| Family Verrucomicrobiaceae | Diabetic kidney disease | rs111862613 | 12 | 130309670 | T | C | 0.1776 | 0.090706497 | 0.0034 | 0.01967463 | 0.0346 | 0.00000402 | 0.9215 | TRUE |
| Family Verrucomicrobiaceae | Diabetic kidney disease | rs117107102 | 18 | 49473635 | A | G | 0.04764 | 0.20468288 | 0.0464 | 0.043157251 | 0.0604 | 0.00000211 | 0.442 | TRUE |
| Family Verrucomicrobiaceae | Diabetic kidney disease | rs11729256 | 4 | 95027272 | T | C | 0.1696 | 0.074979802 | 0.0397 | 0.015017677 | 0.0344 | 0.000000595 | 0.2488 | TRUE |
| Family Verrucomicrobiaceae | Diabetic kidney disease | rs12908520 | 15 | 97570657 | G | A | 0.4268 | 0.061910394 | 0.025 | 0.013094609 | 0.0261 | 0.00000227 | 0.3382 | TRUE |
| Family Verrucomicrobiaceae | Diabetic kidney disease | rs2602429 | 16 | 81063149 | C | T | 0.2612 | 0.074551564 | 0.0904 | 0.015619484 | 0.0295 | 0.00000182 | 0.00216701 | TRUE |
| Family Verrucomicrobiaceae | Diabetic kidney disease | rs34896295 | 9 | 139060991 | A | T | 0.1079 | -0.108532331 | 0.024 | 0.024341603 | 0.0417 | 0.00000825 | 0.5645 | TRUE |
| Family Verrucomicrobiaceae | Diabetic kidney disease | rs4242783 | 10 | 5064327 | G | A | 0.2833 | 0.068799208 | 0.0178 | 0.01476936 | 0.0288 | 0.00000319 | 0.5369 | TRUE |
| Family Verrucomicrobiaceae | Diabetic kidney disease | rs61779207 | 1 | 41074472 | G | A | 0.2236 | -0.075881119 | -0.0447 | 0.01677619 | 0.0311 | 0.00000609 | 0.1508 | TRUE |
| Family Verrucomicrobiaceae | Diabetic kidney disease | rs7100838 | 10 | 60876067 | G | A | 0.1359 | 0.076799608 | 0.0134 | 0.017271377 | 0.0377 | 0.00000872 | 0.7228 | TRUE |
| Family Verrucomicrobiaceae | Diabetic kidney disease | rs72999540 | 2 | 240347720 | T | C | 0.05509 | -0.126942034 | 0.054 | 0.028407992 | 0.0566 | 0.00000788 | 0.34 | TRUE |
| Family Verrucomicrobiaceae | Diabetic kidney disease | rs74542928 | 4 | 100544188 | T | C | 0.04797 | 0.112123956 | 0.0947 | 0.023642178 | 0.0605 | 0.00000211 | 0.1178 | TRUE |
| Family Verrucomicrobiaceae | Diabetic kidney disease | rs9349825 | 6 | 56341481 | A | G | 0.1895 | -0.070435405 | 0.0187 | 0.014712796 | 0.0331 | 0.00000169 | 0.5727 | TRUE |
| Genus Akkermansia | Diabetic kidney disease | rs11184341 | 1 | 105422565 | G | C | 0.2743 | 0.065585479 | -0.0061 | 0.01422364 | 0.0289 | 4.007E-06 | 0.8327 | TRUE |
| Genus Akkermansia | Diabetic kidney disease | rs111862613 | 12 | 130309670 | T | C | 0.1776 | 0.091119928 | 0.0034 | 0.019674815 | 0.0346 | 3.63381E-06 | 0.9215 | TRUE |
| Genus Akkermansia | Diabetic kidney disease | rs117107102 | 18 | 49473635 | A | G | 0.04764 | 0.204406166 | 0.0464 | 0.043162894 | 0.0604 | 2.18309E-06 | 0.442 | TRUE |
| Genus Akkermansia | Diabetic kidney disease | rs11729256 | 4 | 95027272 | T | C | 0.1696 | 0.075047253 | 0.0397 | 0.015018395 | 0.0344 | 5.82224E-07 | 0.2488 | TRUE |
| Genus Akkermansia | Diabetic kidney disease | rs12908520 | 15 | 97570657 | G | A | 0.4268 | 0.061772023 | 0.025 | 0.01309539 | 0.0261 | 2.39252E-06 | 0.3382 | TRUE |
| Genus Akkermansia | Diabetic kidney disease | rs2602429 | 16 | 81063149 | C | T | 0.2612 | 0.074535241 | 0.0904 | 0.015620127 | 0.0295 | 1.82638E-06 | 0.00216701 | TRUE |
| Genus Akkermansia | Diabetic kidney disease | rs34896295 | 9 | 139060991 | A | T | 0.1079 | -0.108411893 | 0.024 | 0.024341631 | 0.0417 | 8.43776E-06 | 0.5645 | TRUE |
| Genus Akkermansia | Diabetic kidney disease | rs4242783 | 10 | 5064327 | G | A | 0.2833 | 0.068545381 | 0.0178 | 0.014770062 | 0.0288 | 3.47009E-06 | 0.5369 | TRUE |
| Genus Akkermansia | Diabetic kidney disease | rs61779207 | 1 | 41074472 | G | A | 0.2236 | -0.076053856 | -0.0447 | 0.01677691 | 0.0311 | 5.80842E-06 | 0.1508 | TRUE |
| Genus Akkermansia | Diabetic kidney disease | rs7100838 | 10 | 60876067 | G | A | 0.1359 | 0.0765167 | 0.0134 | 0.017272258 | 0.0377 | 9.42179E-06 | 0.7228 | TRUE |
| Genus Akkermansia | Diabetic kidney disease | rs72999540 | 2 | 240347720 | T | C | 0.05509 | -0.126737213 | 0.054 | 0.028409305 | 0.0566 | 8.15339E-06 | 0.34 | TRUE |
| Genus Akkermansia | Diabetic kidney disease | rs74542928 | 4 | 100544188 | T | C | 0.04797 | 0.112622737 | 0.0947 | 0.023643438 | 0.0605 | 1.90374E-06 | 0.1178 | TRUE |
| Genus Akkermansia | Diabetic kidney disease | rs9349825 | 6 | 56341481 | A | G | 0.1895 | -0.070340692 | 0.0187 | 0.014713291 | 0.0331 | 1.74635E-06 | 0.5727 | TRUE |
| Genus ErysipelotrichaceaeUCG003 | Diabetic kidney disease | rs11666127 | 19 | 57828980 | A | G | 0.177 | -0.07191871 | -0.0026 | 0.016137058 | 0.034 | 8.32146E-06 | 0.938 | TRUE |
| Genus ErysipelotrichaceaeUCG003 | Diabetic kidney disease | rs11877693 | 18 | 43077293 | C | G | 0.1359 | -0.094836864 | 0.0348 | 0.018922189 | 0.0379 | 5.38842E-07 | 0.358 | TRUE |
| Genus ErysipelotrichaceaeUCG003 | Diabetic kidney disease | rs11994308 | 8 | 91660344 | C | T | 0.08802 | 0.115417413 | -0.0037 | 0.024250252 | 0.0455 | 1.94139E-06 | 0.9358 | TRUE |
| Genus ErysipelotrichaceaeUCG003 | Diabetic kidney disease | rs12251396 | 10 | 873148 | A | G | 0.1534 | -0.070539949 | 0.0011 | 0.015876598 | 0.0359 | 8.87073E-06 | 0.976 | TRUE |
| Genus ErysipelotrichaceaeUCG003 | Diabetic kidney disease | rs17798136 | 7 | 33684926 | G | A | 0.06842 | 0.15881755 | -0.0598 | 0.034767431 | 0.0525 | 4.92405E-06 | 0.2543 | TRUE |
| Genus ErysipelotrichaceaeUCG003 | Diabetic kidney disease | rs2044550 | 18 | 46582100 | T | C | 0.2055 | -0.060934746 | 0.0148 | 0.01360429 | 0.0319 | 7.49643E-06 | 0.643 | TRUE |
| Genus ErysipelotrichaceaeUCG003 | Diabetic kidney disease | rs28568391 | 10 | 125628540 | A | G | 0.5137 | -0.058391081 | 0.0488 | 0.011874052 | 0.026 | 8.76402E-07 | 0.0597806 | TRUE |
| Genus ErysipelotrichaceaeUCG003 | Diabetic kidney disease | rs4758231 | 11 | 7765096 | G | T | 0.2815 | -0.05517544 | 0.0165 | 0.012204675 | 0.0287 | 6.15934E-06 | 0.5659 | TRUE |
| Genus ErysipelotrichaceaeUCG003 | Diabetic kidney disease | rs59068084 | 4 | 113256737 | T | G | 0.3912 | 0.056477243 | 0.024 | 0.012022387 | 0.0265 | 2.63142E-06 | 0.3654 | TRUE |
| Genus ErysipelotrichaceaeUCG003 | Diabetic kidney disease | rs62403464 | 6 | 24220160 | T | C | 0.182 | -0.073174326 | 0.0387 | 0.015651572 | 0.0334 | 2.93659E-06 | 0.2471 | TRUE |
| Genus ErysipelotrichaceaeUCG003 | Diabetic kidney disease | rs6875357 | 5 | 32345671 | C | T | 0.0391 | 0.165673441 | -0.0291 | 0.035383116 | 0.0671 | 2.8371E-06 | 0.664899 | TRUE |
| Genus ErysipelotrichaceaeUCG003 | Diabetic kidney disease | rs75949021 | 2 | 175403951 | T | C | 0.03966 | -0.16966706 | 0.1114 | 0.037423793 | 0.0657 | 5.79681E-06 | 0.0900098 | TRUE |
| Genus ErysipelotrichaceaeUCG003 | Diabetic kidney disease | rs76502207 | 15 | 86522244 | T | C | 0.04017 | 0.14484376 | 0.0108 | 0.028992913 | 0.0662 | 5.85823E-07 | 0.8711 | TRUE |
| Genus ErysipelotrichaceaeUCG003 | Diabetic kidney disease | rs76544074 | 5 | 122804102 | G | C | 0.05598 | -0.121247204 | 0.1307 | 0.02709513 | 0.0559 | 7.64576E-06 | 0.01945 | TRUE |
| Genus ErysipelotrichaceaeUCG003 | Diabetic kidney disease | rs79396538 | 14 | 47935928 | C | G | 0.05631 | 0.084814752 | 0.035 | 0.019114638 | 0.0559 | 9.11525E-06 | 0.531001 | TRUE |
| Genus ErysipelotrichaceaeUCG003 | Diabetic kidney disease | rs8053479 | 16 | 4076830 | A | G | 0.1167 | -0.083821185 | 0.0104 | 0.018650912 | 0.0406 | 6.98275E-06 | 0.798 | TRUE |
| Genus Hungatella | Diabetic kidney disease | rs12095986 | 1 | 178931282 | A | G | 0.09268 | 0.182822571 | 0.0385 | 0.04138542 | 0.0448 | 0.00000998 | 0.3908 | TRUE |
| Genus Hungatella | Diabetic kidney disease | rs13249325 | 8 | 14853525 | T | G | 0.4683 | -0.100022583 | 0.012 | 0.022588331 | 0.0259 | 0.00000951 | 0.6427 | TRUE |
| Genus Hungatella | Diabetic kidney disease | rs17092615 | 14 | 95973619 | G | A | 0.139 | 0.152234676 | 0.032 | 0.033786729 | 0.0373 | 0.00000661 | 0.3912 | TRUE |
| Genus Hungatella | Diabetic kidney disease | rs34471047 | 4 | 166078307 | A | G | 0.1763 | -0.162870912 | -0.0217 | 0.033489966 | 0.0338 | 0.00000115 | 0.5221 | TRUE |
| Genus Hungatella | Diabetic kidney disease | rs62338229 | 4 | 185756720 | C | A | 0.1882 | -0.128494283 | -0.0592 | 0.028999778 | 0.0329 | 0.00000939 | 0.0717993 | TRUE |
| Genus Hungatella | Diabetic kidney disease | rs72759041 | 15 | 89577376 | G | T | 0.209 | -0.126025023 | -0.0438 | 0.028224238 | 0.0319 | 0.000008 | 0.1701 | TRUE |
| Genus Marvinbryantia | Diabetic kidney disease | rs11645029 | 16 | 5938448 | G | C | 0.5034 | -0.060618043 | -0.0224 | 0.013155125 | 0.0259 | 4.06676E-06 | 0.3868 | TRUE |
| Genus Marvinbryantia | Diabetic kidney disease | rs1187983 | 1 | 58441860 | C | T | 0.1035 | -0.093545581 | -0.0734 | 0.019317443 | 0.0425 | 1.28187E-06 | 0.0842597 | TRUE |
| Genus Marvinbryantia | Diabetic kidney disease | rs146541147 | 19 | 4542097 | G | A | 0.03358 | 0.118845391 | 0.1226 | 0.02684231 | 0.0721 | 9.5314E-06 | 0.0888403 | TRUE |
| Genus Marvinbryantia | Diabetic kidney disease | rs17042065 | 2 | 587649 | G | A | 0.07044 | -0.104190198 | -0.0146 | 0.023540699 | 0.0507 | 9.60142E-06 | 0.773701 | TRUE |
| Genus Marvinbryantia | Diabetic kidney disease | rs2724813 | 10 | 12516924 | A | G | 0.2363 | -0.084076924 | -0.0245 | 0.01675508 | 0.0303 | 5.22133E-07 | 0.4187 | TRUE |
| Genus Marvinbryantia | Diabetic kidney disease | rs2842896 | 6 | 132881664 | C | T | 0.586 | -0.0649396 | 0 | 0.013114646 | 0.0263 | 7.35736E-07 | 0.9987 | TRUE |
| Genus Marvinbryantia | Diabetic kidney disease | rs2863363 | 3 | 166039765 | A | G | 0.2447 | 0.063486062 | 0.0127 | 0.013632279 | 0.03 | 3.20789E-06 | 0.673 | TRUE |
| Genus Marvinbryantia | Diabetic kidney disease | rs3125832 | 1 | 211400180 | A | C | 0.2263 | 0.067932264 | 0.0198 | 0.015011987 | 0.0311 | 6.0338E-06 | 0.5233 | TRUE |
| Genus Marvinbryantia | Diabetic kidney disease | rs61884471 | 11 | 45826561 | G | A | 0.1086 | 0.124426313 | -0.0014 | 0.024843116 | 0.0418 | 5.48608E-07 | 0.9738 | TRUE |
| Genus Marvinbryantia | Diabetic kidney disease | rs72948274 | 11 | 79518683 | A | C | 0.06348 | -0.126353989 | -0.0229 | 0.02722118 | 0.053 | 3.45466E-06 | 0.6655 | TRUE |
| Genus.RuminococcaceaeUCG014 | Diabetic kidney disease | rs10791168 | 11 | 131659000 | A | G | 0.1848 | -0.066462357 | 0.0816 | 0.015006876 | 0.0336 | 9.47616E-06 | 0.0152799 | TRUE |
| Genus.RuminococcaceaeUCG014 | Diabetic kidney disease | rs10941294 | 5 | 36435597 | C | T | 0.05447 | -0.122057405 | -0.0193 | 0.026001669 | 0.0568 | 2.67634E-06 | 0.734 | TRUE |
| Genus.RuminococcaceaeUCG014 | Diabetic kidney disease | rs112829527 | 18 | 2371970 | G | A | 0.05739 | 0.120654115 | -0.1755 | 0.026811305 | 0.0563 | 6.79148E-06 | 0.00183 | TRUE |
| Genus.RuminococcaceaeUCG014 | Diabetic kidney disease | rs115777838 | 5 | 26110626 | T | C | 0.1132 | -0.188349525 | 0.0471 | 0.038664295 | 0.041 | 1.10806E-06 | 0.2499 | TRUE |
| Genus.RuminococcaceaeUCG014 | Diabetic kidney disease | rs12638134 | 3 | 101334609 | T | G | 0.4162 | 0.058254795 | 0.0068 | 0.011965732 | 0.0261 | 1.12466E-06 | 0.7954 | TRUE |
| Genus.RuminococcaceaeUCG014 | Diabetic kidney disease | rs17296933 | 19 | 49175964 | C | G | 0.1139 | -0.08295439 | 0.0318 | 0.018505763 | 0.0409 | 7.37305E-06 | 0.4366 | TRUE |
| Genus.RuminococcaceaeUCG014 | Diabetic kidney disease | rs439810 | 2 | 119467367 | G | C | 0.3293 | -0.057712693 | 0.0091 | 0.012667538 | 0.0275 | 5.21489E-06 | 0.7392 | TRUE |
| Genus.RuminococcaceaeUCG014 | Diabetic kidney disease | rs56105232 | 9 | 14363769 | G | A | 0.06234 | 0.139275689 | 0.022 | 0.029913177 | 0.0543 | 3.22415E-06 | 0.685799 | TRUE |
| Genus.RuminococcaceaeUCG014 | Diabetic kidney disease | rs62478832 | 7 | 109564034 | T | A | 0.3405 | -0.058128986 | -0.0256 | 0.012903031 | 0.0273 | 6.63528E-06 | 0.3485 | TRUE |
| Genus.RuminococcaceaeUCG014 | Diabetic kidney disease | rs72809222 | 2 | 57205854 | T | C | 0.2118 | 0.067177506 | 0.0209 | 0.013983796 | 0.0317 | 1.55563E-06 | 0.5106 | TRUE |
| Genus.RuminococcaceaeUCG014 | Diabetic kidney disease | rs73186226 | 3 | 123829361 | G | A | 0.07546 | -0.099385194 | 0.0174 | 0.02168186 | 0.0499 | 4.56613E-06 | 0.726599 | TRUE |
| Genus.RuminococcaceaeUCG014 | Diabetic kidney disease | rs74060145 | 1 | 15322582 | C | G | 0.07906 | -0.115766761 | 0.0002 | 0.025419855 | 0.0479 | 5.25887E-06 | 0.9974 | TRUE |
| Genus.RuminococcaceaeUCG014 | Diabetic kidney disease | rs7528298 | 1 | 116372850 | A | C | 0.02929 | -0.097456455 | 0.0254 | 0.022021038 | 0.0772 | 9.61716E-06 | 0.742 | TRUE |
| Genus.RuminococcaceaeUCG014 | Diabetic kidney disease | rs7655928 | 4 | 95568956 | C | A | 0.159 | -0.065218393 | 0.0294 | 0.014478208 | 0.0353 | 6.65011E-06 | 0.4049 | TRUE |
| Genus.RuminococcaceaeUCG014 | Diabetic kidney disease | rs77627087 | 16 | 87784994 | C | G | 0.1732 | 0.067561994 | -0.0207 | 0.015010339 | 0.034 | 6.76248E-06 | 0.5438 | TRUE |
| Genus.RuminococcaceaeUCG014 | Diabetic kidney disease | rs853612 | 10 | 119928413 | A | G | 0.3953 | -0.05282572 | 0.0021 | 0.011936668 | 0.0263 | 9.62193E-06 | 0.936 | TRUE |
| Genus.RuminococcaceaeUCG014 | Diabetic kidney disease | rs995642 | 2 | 134854659 | C | T | 0.2391 | 0.060047967 | -0.0399 | 0.012641687 | 0.0303 | 2.0342E-06 | 0.1872 | TRUE |

**Table S4 The forward MR results of causal effect of gut microbiota on diabetic kidney disease.**

|  |  |  |  |  |  |  |  |  |  |  | **Horizontal pleiotropy (MR-Egger)** | | | **Heterogeneity （Cochran's Q test)** | | **MR-PRESSO** |
| --- | --- | --- | --- | --- | --- | --- | --- | --- | --- | --- | --- | --- | --- | --- | --- | --- |
| **Exposure** | **Method** | **Nsnp** | **b** | **se** | **OR** | **95%CI_low** | **95%CI_up** | **P valueccccccc** | **P**_adjust_ | **Direction** | **Egger intercept** | **SE** | **P value** | **Q** | **P value** | **P value** |
| Phylum Bacteroidetes |  |  |  |  |  |  |  |  |  |  |  |  |  |  |  |  |
|  | MR Egger | 13 | 0.478998 | 0.300494 | 1.614456 | 0.89586082 | 2.909457517 | 0.13923497 | 0.933569 |  |  |  |  |  |  |  |
|  | Weighted median | 13 | 0.44889 | 0.187096 | 1.566573 | 1.085654107 | 2.260527302 | 0.00917864 | 0.082608 |  |  |  |  |  |  |  |
|  | Inverse variance weight | 13 | 0.279617 | 0.13425 | 1.322623 | 1.016624176 | 1.720727132 | 0.03726836 | 0.165052 | risk | -0.0167 | 0.0225 | 0.4738 | 11.27 | 0.5058 | 0.5382 |
|  | Simple mode | 13 | 0.556392 | 0.2839 | 1.744367 | 0.999947783 | 3.042974335 | 0.08357002 | 0.312079 |  |  |  |  |  |  |  |
|  | Weighted mode | 13 | 0.563288 | 0.256491 | 1.756439 | 1.062437507 | 2.90377279 | 0.04982606 | 0.448435 |  |  |  |  |  |  |  |
| Phylum Verrucomicrobia |  |  |  |  |  |  |  |  |  |  |  |  |  |  |  |  |
|  | MR Egger | 11 | 0.030912 | 0.495647 | 1.031395 | 0.390411116 | 2.724757325 | 0.95163334 | 0.951633 |  |  |  |  |  |  |  |
|  | Weighted median | 11 | 0.34352 | 0.193044 | 1.409901 | 0.965753569 | 2.058311166 | 0.07325814 | 0.329662 |  |  |  |  |  |  |  |
|  | Inverse variance weight | 11 | 0.359152 | 0.162596 | 1.432114 | 1.041293805 | 1.969616936 | 0.0271842 | 0.165052 | risk | 0.0302 | 0.043 | 0.4997 | 15.78 | 0.1061 | 0.1406 |
|  | Simple mode | 11 | 0.561401 | 0.324786 | 1.753126 | 0.927577062 | 3.313418786 | 0.12731545 | 0.312079 |  |  |  |  |  |  |  |
|  | Weighted mode | 11 | 0.375822 | 0.246567 | 1.456188 | 0.898123105 | 2.361017347 | 0.17545964 | 0.526073 |  |  |  |  |  |  |  |
| Class Bacteroidia |  |  |  |  |  |  |  |  |  |  |  |  |  |  |  |  |
|  | MR Egger | 14 | 0.479426 | 0.306243 | 1.615148 | 0.886202619 | 2.943685262 | 0.14344036 | 0.821094 |  |  |  |  |  |  |  |
|  | Weighted median | 14 | 0.506281 | 0.186917 | 1.65911 | 1.150186438 | 2.393217744 | 0.00552774 | 0.088444 |  |  |  |  |  |  |  |
|  | Inverse variance weight | 14 | 0.284391 | 0.136085 | 1.328953 | 1.017822186 | 1.735190618 | 0.03663482 | 0.293079 | risk | -0.0156 | 0.0218 | 0.4887 | 13.59 | 0.4031 | 0.4516 |
|  | Simple mode | 14 | 0.593078 | 0.292645 | 1.80955 | 1.019684522 | 3.21125962 | 0.06256876 | 0.890208 |  |  |  |  |  |  |  |
|  | Weighted mode | 14 | 0.578866 | 0.255416 | 1.784015 | 1.081393836 | 2.943154056 | 0.05818373 | 0.888516 |  |  |  |  |  |  |  |
| Class Verrucomicrobiae |  |  |  |  |  |  |  |  |  |  |  |  |  |  |  |  |
|  | MR Egger | 13 | 0.045705 | 0.425547 | 0.955324 | 0.414875236 | 2.199802198 | 0.88466006 | 0.929229 |  |  |  |  |  |  |  |
|  | Weighted median | 13 | 0.214879 | 0.165702 | 1.239712 | 0.89592588 | 1.715415443 | 0.14775173 | 0.652344 |  |  |  |  |  |  |  |
|  | Inverse variance weight | 13 | 0.246473 | 0.12516 | 1.279505 | 1.001160922 | 1.635234543 | 0.02617146 | 0.293079 | risk | 0.0257 | 0.0357 | 0.4867 | 14.26 | 0.2844 | 0.3061 |
|  | Simple mode | 13 | 0.19257 | 0.283847 | 1.212361 | 0.695051285 | 2.114693222 | 0.29108395 | 0.890208 |  |  |  |  |  |  |  |
|  | Weighted mode | 13 | 0.18642 | 0.240569 | 1.204928 | 0.751943344 | 1.930800513 | 0.31102924 | 0.888516 |  |  |  |  |  |  |  |
| Order Bacteroidales |  |  |  |  |  |  |  |  |  |  |  |  |  |  |  |  |
|  | MR Egger | 14 | 0.479426 | 0.306243 | 1.615148 | 0.886202619 | 2.943685262 | 0.14344036 | 0.670149 |  |  |  |  |  |  |  |
|  | Weighted median | 14 | 0.506281 | 0.178858 | 1.65911 | 1.16849763 | 2.355714313 | 0.0056975 | 0.11395 |  |  |  |  |  |  |  |
|  | Inverse variance weight | 14 | 0.284391 | 0.136085 | 1.328953 | 1.017822186 | 1.735190618 | 0.03663482 | 0.366348 | risk | -0.0156 | 0.0218 | 0.4887 | 13.59 | 0.4031 | 0.4481 |
|  | Simple mode | 14 | 0.593078 | 0.279765 | 1.80955 | 1.045755394 | 3.131202336 | 0.07112579 | 0.80624 |  |  |  |  |  |  |  |
|  | Weighted mode | 14 | 0.578866 | 0.253788 | 1.784015 | 1.084850289 | 2.93377684 | 0.03613318 | 0.722664 |  |  |  |  |  |  |  |
| Order Verrucomicrobiales |  |  |  |  |  |  |  |  |  |  |  |  |  |  |  |  |
|  | MR Egger | 13 | 0.045705 | 0.425547 | 0.955324 | 0.414875236 | 2.199802198 | 0.88466006 | 0.930352 |  |  |  |  |  |  |  |
|  | Weighted median | 13 | 0.214879 | 0.161855 | 1.239712 | 0.902707427 | 1.702528465 | 0.13757774 | 0.687889 |  |  |  |  |  |  |  |
|  | Inverse variance weight | 13 | 0.246473 | 0.12516 | 1.279505 | 1.001160922 | 1.635234543 | 0.02617146 | 0.366348 | risk | 0.0273 | 0.03278 | 0.4205 | 14.26 | 0.2844 | 0.3782 |
|  | Simple mode | 13 | 0.19257 | 0.260143 | 1.212361 | 0.728104739 | 2.018693415 | 0.32934857 | 0.80624 |  |  |  |  |  |  |  |
|  | Weighted mode | 13 | 0.18642 | 0.243061 | 1.204928 | 0.748279343 | 1.940254807 | 0.30879149 | 0.884272 |  |  |  |  |  |  |  |
| Family Peptostreptococcaceae |  |  |  |  |  |  |  |  |  |  |  |  |  |  |  |  |
|  | MR Egger | 15 | 0.273518 | 0.260649 | 1.31458 | 0.788712199 | 2.191067446 | 0.31312432 | 0.879252 |  |  |  |  |  |  |  |
|  | Weighted median | 15 | 0.384436 | 0.159852 | 1.468785 | 1.073716858 | 2.009216268 | 0.01321975 | 0.423032 |  |  |  |  |  |  |  |
|  | Inverse variance weight | 15 | 0.332613 | 0.108751 | 1.394607 | 1.126888575 | 1.725929186 | 0.00222468 | 0.07119 | risk | 0.0051 | 0.0202 | 0.8057 | 13.87 | 0.4596 | 0.5134 |
|  | Simple mode | 15 | 0.498902 | 0.253768 | 1.646912 | 1.001517762 | 2.708209651 | 0.07009884 | 0.841396 |  |  |  |  |  |  |  |
|  | Weighted mode | 15 | 0.397054 | 0.223935 | 1.487436 | 0.959006062 | 2.307041153 | 0.08489017 | 0.964885 |  |  |  |  |  |  |  |
| Family Verrucomicrobiaceae |  |  |  |  |  |  |  |  |  |  |  |  |  |  |  |  |
|  | MR Egger | 13 | 0.047396 | 0.425406 | 0.95371 | 0.414288645 | 2.195478974 | 0.88150008 | 0.969665 |  |  |  |  |  |  |  |
|  | Weighted median | 13 | 0.214711 | 0.160473 | 1.239503 | 0.905003347 | 1.697638928 | 0.13682059 | 0.875652 |  |  |  |  |  |  |  |
|  | Inverse variance weight | 13 | 0.246346 | 0.125207 | 1.279342 | 1.000940476 | 1.635177354 | 0.02629588 | 0.420734 | risk | 0.0258 | 0.0357 | 0.4842 | 14.27 | 0.284 | 0.3139 |
|  | Simple mode | 13 | 0.193527 | 0.247703 | 1.213522 | 0.746790331 | 1.971953891 | 0.31094749 | 0.841396 |  |  |  |  |  |  |  |
|  | Weighted mode | 13 | 0.187371 | 0.231773 | 1.206075 | 0.765745892 | 1.899606966 | 0.29383263 | 0.964885 |  |  |  |  |  |  |  |
| Genus Akkermansia |  |  |  |  |  |  |  |  |  |  |  |  |  |  |  |  |
|  | MR Egger | 13 | 0.04483 | 0.425397 | 0.95616 | 0.415360644 | 2.201081947 | 0.88639469 | 0.947681 |  |  |  |  |  |  |  |
|  | Weighted median | 13 | 0.215042 | 0.170212 | 1.239913 | 0.888186805 | 1.730925542 | 0.13551287 | 0.992676 |  |  |  |  |  |  |  |
|  | Inverse variance weight | 13 | 0.246628 | 0.125201 | 1.279703 | 1.001234191 | 1.635620318 | 0.02612121 | 0.922033 | risk | 0.0256 | 0.0357 | 0.4875 | 14.26 | 0.2845 | 0.3141 |
|  | Simple mode | 13 | 0.193317 | 0.268446 | 1.213267 | 0.716887572 | 2.05334519 | 0.29800152 | 0.999212 |  |  |  |  |  |  |  |
|  | Weighted mode | 13 | 0.187161 | 0.22907 | 1.205821 | 0.769652941 | 1.889170239 | 0.32480991 | 0.992312 |  |  |  |  |  |  |  |
| Genus Erysipelotrichaceae UCG003 | |  |  |  |  |  |  |  |  |  |  |  |  |  |  |  |
|  | MR Egger | 16 | 0.456659 | 0.304667 | 0.633396 | 0.348608181 | 1.150836017 | 0.15611304 | 0.947681 |  |  |  |  |  |  |  |
|  | Weighted median | 16 | 0.245236 | 0.153871 | 0.78252 | 0.578786242 | 1.057968249 | 0.11951298 | 0.992676 |  |  |  |  |  |  |  |
|  | Inverse variance weight | 16 | 0.277527 | 0.111294 | 0.757655 | 0.609166943 | 0.942338104 | 0.01264397 | 0.922033 | protective | 0.0164 | 0.026 | 0.5378 | 11.18 | 0.7397 | 0.7517 |
|  | Simple mode | 16 | 0.15688 | 0.267795 | 0.854806 | 0.505726526 | 1.444839558 | 0.57031075 | 0.999212 |  |  |  |  |  |  |  |
|  | Weighted mode | 16 | 0.201131 | 0.243255 | 0.817805 | 0.507676292 | 1.317386382 | 0.39993445 | 0.992312 |  |  |  |  |  |  |  |
| Genus Hungatella |  |  |  |  |  |  |  |  |  |  |  |  |  |  |  |  |
|  | MR Egger | 6 | 0.476396 | 0.502037 | 1.610261 | 0.601940419 | 4.307635095 | 0.39639191 | 0.947681 |  |  |  |  |  |  |  |
|  | Weighted median | 6 | 0.210272 | 0.126295 | 1.234013 | 0.963419169 | 1.580608432 | 0.08430919 | 0.992676 |  |  |  |  |  |  |  |
|  | Inverse variance weight | 6 | 0.202969 | 0.098867 | 1.225034 | 1.009232322 | 1.486980314 | 0.04007713 | 0.922033 | risk | -0.0381 | 0.0685 | 0.6081 | 3.01 | 0.6985 | 0.7431 |
|  | Simple mode | 6 | 0.215412 | 0.188957 | 1.240373 | 0.856463737 | 1.796368494 | 0.30480926 | 0.999212 |  |  |  |  |  |  |  |
|  | Weighted mode | 6 | 0.196935 | 0.184056 | 1.217665 | 0.848898604 | 1.74662466 | 0.31099275 | 0.992312 |  |  |  |  |  |  |  |
| Genus Marvinbryantia |  |  |  |  |  |  |  |  |  |  |  |  |  |  |  |  |
|  | MR Egger | 10 | 0.298482 | 0.50901 | 1.347812 | 0.496993794 | 3.655169085 | 0.57377759 | 0.947681 |  |  |  |  |  |  |  |
|  | Weighted median | 10 | 0.198818 | 0.184327 | 1.21996 | 0.850046789 | 1.750847062 | 0.26949841 | 0.992676 |  |  |  |  |  |  |  |
|  | Inverse variance weight | 10 | 0.267638 | 0.13502 | 1.306874 | 1.003002596 | 1.702806727 | 0.04745633 | 0.922033 | risk | -0.0026 | 0.0415 | 0.9514 | 4.2021 | 0.8976 | 0.91 |
|  | Simple mode | 10 | 0.22604 | 0.253348 | 1.253625 | 0.762980976 | 2.059784384 | 0.42249235 | 0.999212 |  |  |  |  |  |  |  |
|  | Weighted mode | 10 | 0.238738 | 0.230699 | 1.269646 | 0.80780706 | 1.995527312 | 0.32810788 | 0.992312 |  |  |  |  |  |  |  |
| Genus RuminococcaceaeUCG014 | |  |  |  |  |  |  |  |  |  |  |  |  |  |  |  |
|  | MR Egger | 17 | 0.307969 | 0.292665 | 0.734938 | 0.414123034 | 1.304283926 | 0.30932283 | 0.947681 |  |  |  |  |  |  |  |
|  | Weighted median | 17 | 0.185754 | 0.151971 | 0.830478 | 0.616549721 | 1.118633944 | 0.23482566 | 0.992676 |  |  |  |  |  |  |  |
|  | Inverse variance weight | 17 | 0.221272 | 0.111569 | 0.801499 | 0.644070491 | 0.997406154 | 0.04733627 | 0.922033 | protective | 0.0077 | 0.024 | 0.7519 | 17.9356 | 0.3277 | 0.3642 |
|  | Simple mode | 17 | 0.152914 | 0.236927 | 0.858204 | 0.539403499 | 1.365422227 | 0.53393486 | 0.999212 |  |  |  |  |  |  |  |
|  | Weighted mode | 17 | 0.171392 | 0.187905 | 0.842491 | 0.582931496 | 1.217623329 | 0.3891767 | 0.992312 |  |  |  |  |  |  |  |

**Table S5 The forward MR results of causal effect of gut microbiota on four traits associated with diabetic kidney disease.**

| **c** | **Outcome** | **nSNP** | **MR_Egger** | **Weighted_median** | **Inverse_variance_weighted** | **Simple_mode** | **Weighted_mode** |
| --- | --- | --- | --- | --- | --- | --- | --- |
| Class Actinobacteria | Glomerular filtration rate in diabetics (creatinine) | 10ccc | 0.414123 | 0.036902 | 0.009288 | 0.080006 | 0.087123 |
| Genus Eubacteriumruminantium group | Glomerular filtration rate in diabetics (creatinine) | 9 | 0.638445 | 0.235384 | 0.046673 | 0.894479 | 0.842913 |
| Genus Allisonella | Glomerular filtration rate in diabetics (creatinine) | 3 | 0.6014 | 0.072633 | 0.04692 | 0.21576 | 0.252819 |
| Genus Anaerostipes | Glomerular filtration rate in diabetics (creatinine) | 3 | 0.65994 | 0.059975 | 0.035626 | 0.21281 | 0.220622 |
| Genus Erysipelotrichaceae UCG003 | Glomerular filtration rate in diabetics (creatinine) | 4 | 0.193261 | 0.072216 | 0.047432 | 0.233467 | 0.195333 |
| Genus Ruminococcaceae UCG003 | Glomerular filtration rate in diabetics (creatinine) | 7 | 0.678879 | 0.019707 | 0.026282 | 0.11284 | 0.106974 |
| Genus Ruminococcaceae UCG005 | Glomerular filtration rate in diabetics (creatinine) | 5 | 0.889792 | 0.01118 | 0.030102 | 0.124995 | 0.12346 |
| Class Deltaproteobacteria | UACR in diabetics | 9 | 0.846928 | 0.387288 | 0.046938 | 0.677445 | 0.685569 |
| Family Alcaligenaceae | UACR in diabetics | 6 | 0.599545 | 0.034313 | 0.03103 | 0.146539 | 0.147536 |
| Family Desulfovibrionaceae | UACR in diabetics | 10 | 0.491025 | 0.268513 | 0.034828 | 0.554895 | 0.555804 |
| Genus Ruminococcaceae UCG014 | UACR in diabetics | 7 | 0.720925 | 0.056956 | 0.039421 | 0.153581 | 0.151873 |
| Class Lentisphaeria | Type 1 diabetes with renal complications | 14 | 0.941206 | 0.583119 | 0.030003 | 0.895553 | 0.909992 |
| Order Victivallales | Type 1 diabetes with renal complications | 14 | 0.941206 | 0.5796 | 0.030003 | 0.895203 | 0.91911 |
| Family Peptococcaceae | Type 1 diabetes with renal complications | 10 | 0.919916 | 0.207 | 0.022458 | 0.735675 | 0.837759 |
| Genus Eubacteriumcoprostanoligenes group | Type 1 diabetes with renal complications | 14 | 0.348725 | 0.025379 | 0.024088 | 0.111031 | 0.125104 |
| Genus Catenibacterium | Type 1 diabetes with renal complications | 5 | 0.120877 | 0.028462 | 0.007524 | 0.120072 | 0.125419 |
| Genus Phascolarctobacterium | Type 1 diabetes with renal complications | 12 | 0.244307 | 0.524305 | 0.029038 | 0.838987 | 0.946943 |
| Phylum Bacteroidetes | Type 2 diabetes with renal complications | 13 | 0.803944 | 0.399861 | 0.026684 | 0.819806 | 0.859203 |
| Phylum Lentisphaerae | Type 2 diabetes with renal complications | 14 | 0.967567 | 0.125085 | 0.035278 | 0.490945 | 0.667032 |
| Phylum Proteobacteria | Type 2 diabetes with renal complications | 12 | 0.579005 | 0.082779 | 0.030687 | 0.258513 | 0.281533 |
| Phylum Verrucomicrobia | Type 2 diabetes with renal complications | 11 | 0.96169 | 0.100863 | 0.023818 | 0.233272 | 0.212108 |
| Class Bacteroidia | Type 2 diabetes with renal complications | 14 | 0.74104 | 0.319997 | 0.01787 | 0.537727 | 0.704445 |
| Class Lentisphaeria | Type 2 diabetes with renal complications | 14 | 0.887775 | 0.104013 | 0.048629 | 0.341774 | 0.391417 |
| Class Verrucomicrobiae | Type 2 diabetes with renal complications | 14 | 0.952195 | 0.105355 | 0.023555 | 0.346506 | 0.281098 |
| Order Bacteroidales | Type 2 diabetes with renal complications | 14 | 0.74104 | 0.337351 | 0.01787 | 0.547086 | 0.706728 |
| Order Enterobacteriales | Type 2 diabetes with renal complications | 10 | 0.853801 | 0.085068 | 0.033732 | 0.222007 | 0.224734 |
| Order Verrucomicrobiales | Type 2 diabetes with renal complications | 14 | 0.952195 | 0.112274 | 0.023555 | 0.306575 | 0.322759 |
| Order Victivallales | Type 2 diabetes with renal complications | 14 | 0.887775 | 0.107341 | 0.048629 | 0.369927 | 0.381596 |
| Family Enterobacteriaceae | Type 2 diabetes with renal complications | 10 | 0.853801 | 0.072478 | 0.033732 | 0.195717 | 0.218155 |
| Family Peptostreptococcaceae | Type 2 diabetes with renal complications | 15 | 0.866052 | 0.017786 | 0.032018 | 0.273699 | 0.366755 |
| Family Prevotellaceae | Type 2 diabetes with renal complications | 19 | 0.534531 | 0.865339 | 0.041808 | 0.688045 | 0.657487 |
| Family Verrucomicrobiaceae | Type 2 diabetes with renal complications | 14 | 0.948576 | 0.110714 | 0.023646 | 0.323209 | 0.285068 |
| Genus Akkermansia | Type 2 diabetes with renal complications | 14 | 0.951977 | 0.114838 | 0.023567 | 0.340754 | 0.286023 |
| Genus Erysipelotrichaceae UCG003 | Type 2 diabetes with renal complications | 16 | 0.464578 | 0.061961 | 0.037922 | 0.398499 | 0.327778 |
| Genus Flavonifractor | Type 2 diabetes with renal complications | 9 | 0.864302 | 0.033071 | 0.010221 | 0.154744 | 0.150235 |
| Genus RuminococcaceaeUCG002 | Type 2 diabetes with renal complications | 26 | 0.368805 | 0.223691 | 0.028163 | 0.780975 | 0.931742 |

**Table S6 Information about the selected SNPs of the reverse MR analysis.**

| **Exposurecccccccccccccc** | **Outcome** | **SNP** | **Chr** | **Position** | **Effect allele** | **Other allele** | **MAF** | **Beta.exposure** | **Beta.outcome** | **SE.exposure** | **SE.outcome** | **P-value.exposure** | **P-value.outcome** | **mr_keep** |
| --- | --- | --- | --- | --- | --- | --- | --- | --- | --- | --- | --- | --- | --- | --- |
| Diabetic kidney disease | Phylum Actinobacteria | rs10489621 | 1 | 57238528 | C | T | 0.12 | 0.1743 | -0.00093427 | 0.0394 | 0.015940966 | 9.92202E-06 | 0.953264251 | TRUE |
| Diabetic kidney disease | Phylum Actinobacteria | rs10507642 | 13 | 60481883 | C | T | 0.119 | 0.1801 | -0.005900254 | 0.0399 | 0.017952526 | 6.24195E-06 | 0.742413652 | TRUE |
| Diabetic kidney disease | Phylum Actinobacteria | rs111366116 | 5 | 53295546 | T | C | 0.1207 | 0.1789 | -0.009080782 | 0.0396 | 0.017755966 | 6.19398E-06 | 0.609055961 | TRUE |
| Diabetic kidney disease | Phylum Actinobacteria | rs12505710 | 4 | 8565712 | A | G | 0.1449 | -0.1754 | 0.020533711 | 0.0385 | 0.019203501 | 5.29298E-06 | 0.284948409 | TRUE |
| Diabetic kidney disease | Phylum Actinobacteria | rs2471672 | 15 | 36531651 | A | G | 0.7697 | -0.136 | 0.011770308 | 0.0306 | 0.013449561 | 8.9929E-06 | 0.381495352 | TRUE |
| Diabetic kidney disease | Phylum Actinobacteria | rs2476601 | 1 | 114377568 | G | A | 0.8529 | -0.1765 | 0.020392305 | 0.0361 | 0.018500132 | 1.04201E-06 | 0.270340451 | TRUE |
| Diabetic kidney disease | Phylum Actinobacteria | rs2852217 | 11 | 120647226 | T | C | 0.7149 | 0.1356 | -0.003055228 | 0.0286 | 0.012159058 | 2.13501E-06 | 0.801603998 | TRUE |
| Diabetic kidney disease | Phylum Actinobacteria | rs34771920 | 3 | 29816198 | T | A | 0.0887 | 0.2179 | -0.004529615 | 0.0462 | 0.018184344 | 2.38002E-06 | 0.803287902 | TRUE |
| Diabetic kidney disease | Phylum Actinobacteria | rs34872471 | 10 | 114754071 | C | T | 0.1963 | 0.1551 | -0.03570228 | 0.0326 | 0.01184721 | 1.93999E-06 | 0.002582018 | TRUE |
| Diabetic kidney disease | Phylum Actinobacteria | rs3957146 | 6 | 32681530 | C | T | 0.1149 | 0.7266 | -0.009290081 | 0.0426 | 0.0171745 | 4.43404E-65 | 0.58856069 | TRUE |
| Diabetic kidney disease | Phylum Actinobacteria | rs55694679 | 2 | 238873308 | T | C | 0.06447 | -0.2388 | 0.051275836 | 0.0538 | 0.027492487 | 8.96396E-06 | 0.06216944 | TRUE |
| Diabetic kidney disease | Phylum Actinobacteria | rs76817227 | 3 | 68270049 | T | C | 0.02721 | 0.3551 | 0.002070693 | 0.0803 | 0.030063443 | 9.72098E-06 | 0.945087168 | TRUE |
| Diabetic kidney disease | Phylum Actinobacteria | rs7742705 | 6 | 145001253 | A | G | 0.2213 | 0.1442 | 0.0022852 | 0.0312 | 0.012058971 | 3.72598E-06 | 0.849699301 | TRUE |
| Diabetic kidney disease | Phylum Actinobacteria | rs78670164 | 12 | 12663634 | G | A | 0.01907 | 0.4202 | -0.031954015 | 0.0949 | 0.033772554 | 9.51196E-06 | 0.344070376 | TRUE |
| Diabetic kidney disease | Phylum Proteobacteria | rs10489621 | 1 | 57238528 | C | T | 0.12 | 0.1743 | 0.009199267 | 0.0394 | 0.015842826 | 9.92202E-06 | 0.561470816 | TRUE |
| Diabetic kidney disease | Phylum Proteobacteria | rs10507642 | 13 | 60481883 | C | T | 0.119 | 0.1801 | 0.046396844 | 0.0399 | 0.017801318 | 6.24195E-06 | 0.00915072 | TRUE |
| Diabetic kidney disease | Phylum Proteobacteria | rs111366116 | 5 | 53295546 | T | C | 0.1207 | 0.1789 | 0.030943498 | 0.0396 | 0.017657129 | 6.19398E-06 | 0.079693915 | TRUE |
| Diabetic kidney disease | Phylum Proteobacteria | rs12505710 | 4 | 8565712 | A | G | 0.1449 | -0.1754 | 0.000193355 | 0.0385 | 0.01922555 | 5.29298E-06 | 0.99197565 | TRUE |
| Diabetic kidney disease | Phylum Proteobacteria | rs2471672 | 15 | 36531651 | A | G | 0.7697 | -0.136 | -0.00705924 | 0.0306 | 0.01336888 | 8.9929E-06 | 0.597474846 | TRUE |
| Diabetic kidney disease | Phylum Proteobacteria | rs2476601 | 1 | 114377568 | G | A | 0.8529 | -0.1765 | 0.007008059 | 0.0361 | 0.018524513 | 1.04201E-06 | 0.705198299 | TRUE |
| Diabetic kidney disease | Phylum Proteobacteria | rs2852217 | 11 | 120647226 | T | C | 0.7149 | 0.1356 | 0.007364577 | 0.0286 | 0.012160917 | 2.13501E-06 | 0.544784449 | TRUE |
| Diabetic kidney disease | Phylum Proteobacteria | rs34771920 | 3 | 29816198 | T | A | 0.0887 | 0.2179 | -0.018864045 | 0.0462 | 0.018204587 | 2.38002E-06 | 0.30009725 | TRUE |
| Diabetic kidney disease | Phylum Proteobacteria | rs34872471 | 10 | 114754071 | C | T | 0.1963 | 0.1551 | 0.002074223 | 0.0326 | 0.011756979 | 1.93999E-06 | 0.859960178 | TRUE |
| Diabetic kidney disease | Phylum Proteobacteria | rs3957146 | 6 | 32681530 | C | T | 0.1149 | 0.7266 | 0.036040986 | 0.0426 | 0.016993363 | 4.43404E-65 | 0.033931398 | TRUE |
| Diabetic kidney disease | Phylum Proteobacteria | rs55694679 | 2 | 238873308 | T | C | 0.06447 | -0.2388 | -0.045306089 | 0.0538 | 0.027098087 | 8.96396E-06 | 0.094538236 | TRUE |
| Diabetic kidney disease | Phylum Proteobacteria | rs76817227 | 3 | 68270049 | T | C | 0.02721 | 0.3551 | 0.025034523 | 0.0803 | 0.029569012 | 9.72098E-06 | 0.397191764 | TRUE |
| Diabetic kidney disease | Phylum Proteobacteria | rs7742705 | 6 | 145001253 | A | G | 0.2213 | 0.1442 | 0.010268268 | 0.0312 | 0.011950599 | 3.72598E-06 | 0.390215755 | TRUE |
| Diabetic kidney disease | Phylum Proteobacteria | rs78670164 | 12 | 12663634 | G | A | 0.01907 | 0.4202 | -0.015327637 | 0.0949 | 0.033784568 | 9.51196E-06 | 0.650053739 | TRUE |
| Diabetic kidney disease | Class Alphaproteobacteria | rs10489621 | 1 | 57238528 | C | T | 0.12 | 0.1743 | 0.01805578 | 0.0394 | 0.022365217 | 9.92202E-06 | 0.419484905 | TRUE |
| Diabetic kidney disease | Class Alphaproteobacteria | rs10507642 | 13 | 60481883 | C | T | 0.119 | 0.1801 | 0.028337452 | 0.0399 | 0.02441395 | 6.24195E-06 | 0.245760915 | TRUE |
| Diabetic kidney disease | Class Alphaproteobacteria | rs111366116 | 5 | 53295546 | T | C | 0.1207 | 0.1789 | 0.05926351 | 0.0396 | 0.023806446 | 6.19398E-06 | 0.012796279 | TRUE |
| Diabetic kidney disease | Class Alphaproteobacteria | rs12505710 | 4 | 8565712 | A | G | 0.1449 | -0.1754 | -0.052371243 | 0.0385 | 0.025750711 | 5.29298E-06 | 0.041973935 | TRUE |
| Diabetic kidney disease | Class Alphaproteobacteria | rs2471672 | 15 | 36531651 | A | G | 0.7697 | -0.136 | 0.014023253 | 0.0306 | 0.018712134 | 8.9929E-06 | 0.453603923 | TRUE |
| Diabetic kidney disease | Class Alphaproteobacteria | rs2476601 | 1 | 114377568 | G | A | 0.8529 | -0.1765 | -0.020058078 | 0.0361 | 0.024886541 | 1.04201E-06 | 0.420253831 | TRUE |
| Diabetic kidney disease | Class Alphaproteobacteria | rs2852217 | 11 | 120647226 | T | C | 0.7149 | 0.1356 | 0.01674936 | 0.0286 | 0.0169688 | 2.13501E-06 | 0.323609286 | TRUE |
| Diabetic kidney disease | Class Alphaproteobacteria | rs34771920 | 3 | 29816198 | T | A | 0.0887 | 0.2179 | 0.019744482 | 0.0462 | 0.02497127 | 2.38002E-06 | 0.429126123 | TRUE |
| Diabetic kidney disease | Class Alphaproteobacteria | rs34872471 | 10 | 114754071 | C | T | 0.1963 | 0.1551 | 0.027906288 | 0.0326 | 0.016181221 | 1.93999E-06 | 0.084597871 | TRUE |
| Diabetic kidney disease | Class Alphaproteobacteria | rs3957146 | 6 | 32681530 | C | T | 0.1149 | 0.7266 | 0.036330211 | 0.0426 | 0.023486798 | 4.43404E-65 | 0.121902932 | TRUE |
| Diabetic kidney disease | Class Alphaproteobacteria | rs55694679 | 2 | 238873308 | T | C | 0.06447 | -0.2388 | -0.021279676 | 0.0538 | 0.038234871 | 8.96396E-06 | 0.577833876 | TRUE |
| Diabetic kidney disease | Class Alphaproteobacteria | rs76817227 | 3 | 68270049 | T | C | 0.02721 | 0.3551 | -0.013315136 | 0.0803 | 0.041202099 | 9.72098E-06 | 0.746569184 | TRUE |
| Diabetic kidney disease | Class Alphaproteobacteria | rs7742705 | 6 | 145001253 | A | G | 0.2213 | 0.1442 | 0.001865927 | 0.0312 | 0.016629341 | 3.72598E-06 | 0.91065937 | TRUE |
| Diabetic kidney disease | Class Alphaproteobacteria | rs78670164 | 12 | 12663634 | G | A | 0.01907 | 0.4202 | -0.023709884 | 0.0949 | 0.044699811 | 9.51196E-06 | 0.595817499 | TRUE |
| Diabetic kidney disease | Class Betaproteobacteria | rs10489621 | 1 | 57238528 | C | T | 0.12 | 0.1743 | 0.023772861 | 0.0394 | 0.016349585 | 9.92202E-06 | 0.145936742 | TRUE |
| Diabetic kidney disease | Class Betaproteobacteria | rs10507642 | 13 | 60481883 | C | T | 0.119 | 0.1801 | 0.028772769 | 0.0399 | 0.018381818 | 6.24195E-06 | 0.117516283 | TRUE |
| Diabetic kidney disease | Class Betaproteobacteria | rs111366116 | 5 | 53295546 | T | C | 0.1207 | 0.1789 | 0.04313987 | 0.0396 | 0.018209163 | 6.19398E-06 | 0.017829998 | TRUE |
| Diabetic kidney disease | Class Betaproteobacteria | rs12505710 | 4 | 8565712 | A | G | 0.1449 | -0.1754 | 0.020702122 | 0.0385 | 0.019811339 | 5.29298E-06 | 0.296039942 | TRUE |
| Diabetic kidney disease | Class Betaproteobacteria | rs2471672 | 15 | 36531651 | A | G | 0.7697 | -0.136 | -0.017619893 | 0.0306 | 0.013777694 | 8.9929E-06 | 0.200942483 | TRUE |
| Diabetic kidney disease | Class Betaproteobacteria | rs2476601 | 1 | 114377568 | G | A | 0.8529 | -0.1765 | -0.002035024 | 0.0361 | 0.019092858 | 1.04201E-06 | 0.915117729 | TRUE |
| Diabetic kidney disease | Class Betaproteobacteria | rs2852217 | 11 | 120647226 | T | C | 0.7149 | 0.1356 | 0.012604896 | 0.0286 | 0.012541119 | 2.13501E-06 | 0.314855717 | TRUE |
| Diabetic kidney disease | Class Betaproteobacteria | rs34771920 | 3 | 29816198 | T | A | 0.0887 | 0.2179 | -0.014787608 | 0.0462 | 0.018778459 | 2.38002E-06 | 0.431002573 | TRUE |
| Diabetic kidney disease | Class Betaproteobacteria | rs34872471 | 10 | 114754071 | C | T | 0.1963 | 0.1551 | 0.014135328 | 0.0326 | 0.012130861 | 1.93999E-06 | 0.243923072 | TRUE |
| Diabetic kidney disease | Class Betaproteobacteria | rs3957146 | 6 | 32681530 | C | T | 0.1149 | 0.7266 | 0.015667695 | 0.0426 | 0.017547395 | 4.43404E-65 | 0.371922151 | TRUE |
| Diabetic kidney disease | Class Betaproteobacteria | rs55694679 | 2 | 238873308 | T | C | 0.06447 | -0.2388 | -0.029209881 | 0.0538 | 0.027786469 | 8.96396E-06 | 0.29315444 | TRUE |
| Diabetic kidney disease | Class Betaproteobacteria | rs76817227 | 3 | 68270049 | T | C | 0.02721 | 0.3551 | 0.006232919 | 0.0803 | 0.030634579 | 9.72098E-06 | 0.838775306 | TRUE |
| Diabetic kidney disease | Class Betaproteobacteria | rs7742705 | 6 | 145001253 | A | G | 0.2213 | 0.1442 | -0.014219732 | 0.0312 | 0.012326898 | 3.72598E-06 | 0.248683398 | TRUE |
| Diabetic kidney disease | Class Betaproteobacteria | rs78670164 | 12 | 12663634 | G | A | 0.01907 | 0.4202 | 0.032348487 | 0.0949 | 0.03473263 | 9.51196E-06 | 0.351668792 | TRUE |
| Diabetic kidney disease | Order Rhodospirillales | rs10489621 | 1 | 57238528 | C | T | 0.12 | 0.1743 | 0.030365773 | 0.0394 | 0.023664057 | 9.92202E-06 | 0.1994212 | TRUE |
| Diabetic kidney disease | Order Rhodospirillales | rs10507642 | 13 | 60481883 | C | T | 0.119 | 0.1801 | 0.022589714 | 0.0399 | 0.025742193 | 6.24195E-06 | 0.3801953 | TRUE |
| Diabetic kidney disease | Order Rhodospirillales | rs111366116 | 5 | 53295546 | T | C | 0.1207 | 0.1789 | 0.050847668 | 0.0396 | 0.02515908 | 6.19398E-06 | 0.043274966 | TRUE |
| Diabetic kidney disease | Order Rhodospirillales | rs12505710 | 4 | 8565712 | A | G | 0.1449 | -0.1754 | -0.042989541 | 0.0385 | 0.027461514 | 5.29298E-06 | 0.11747817 | TRUE |
| Diabetic kidney disease | Order Rhodospirillales | rs2471672 | 15 | 36531651 | A | G | 0.7697 | -0.136 | 0.008007207 | 0.0306 | 0.019819986 | 8.9929E-06 | 0.686215213 | TRUE |
| Diabetic kidney disease | Order Rhodospirillales | rs2476601 | 1 | 114377568 | G | A | 0.8529 | -0.1765 | -0.023385482 | 0.0361 | 0.026343624 | 1.04201E-06 | 0.374697108 | TRUE |
| Diabetic kidney disease | Order Rhodospirillales | rs2852217 | 11 | 120647226 | T | C | 0.7149 | 0.1356 | 0.000791786 | 0.0286 | 0.01797628 | 2.13501E-06 | 0.964867598 | TRUE |
| Diabetic kidney disease | Order Rhodospirillales | rs34771920 | 3 | 29816198 | T | A | 0.0887 | 0.2179 | -0.001447895 | 0.0462 | 0.026363481 | 2.38002E-06 | 0.956201827 | TRUE |
| Diabetic kidney disease | Order Rhodospirillales | rs34872471 | 10 | 114754071 | C | T | 0.1963 | 0.1551 | 0.018179699 | 0.0326 | 0.017021367 | 1.93999E-06 | 0.2854972 | TRUE |
| Diabetic kidney disease | Order Rhodospirillales | rs3957146 | 6 | 32681530 | C | T | 0.1149 | 0.7266 | 0.043158982 | 0.0426 | 0.024858305 | 4.43404E-65 | 0.082528524 | TRUE |
| Diabetic kidney disease | Order Rhodospirillales | rs55694679 | 2 | 238873308 | T | C | 0.06447 | -0.2388 | -0.06172361 | 0.0538 | 0.040692395 | 8.96396E-06 | 0.12930859 | TRUE |
| Diabetic kidney disease | Order Rhodospirillales | rs76817227 | 3 | 68270049 | T | C | 0.02721 | 0.3551 | -0.018867876 | 0.0803 | 0.043020541 | 9.72098E-06 | 0.660967127 | TRUE |
| Diabetic kidney disease | Order Rhodospirillales | rs7742705 | 6 | 145001253 | A | G | 0.2213 | 0.1442 | -0.005827688 | 0.0312 | 0.017552528 | 3.72598E-06 | 0.739878557 | TRUE |
| Diabetic kidney disease | Order Rhodospirillales | rs78670164 | 12 | 12663634 | G | A | 0.01907 | 0.4202 | -0.029710443 | 0.0949 | 0.046430167 | 9.51196E-06 | 0.522240716 | TRUE |
| Diabetic kidney disease | Family Bacteroidales S24.7group | rs10489621 | 1 | 57238528 | C | T | 0.12 | 0.1743 | 0.00240189 | 0.0394 | 0.024103409 | 9.92202E-06 | 0.920622674 | TRUE |
| Diabetic kidney disease | Family Bacteroidales S24.7group | rs10507642 | 13 | 60481883 | C | T | 0.119 | 0.1801 | 0.045766128 | 0.0399 | 0.026867509 | 6.24195E-06 | 0.08849315 | TRUE |
| Diabetic kidney disease | Family Bacteroidales S24.7group | rs111366116 | 5 | 53295546 | T | C | 0.1207 | 0.1789 | 0.037207965 | 0.0396 | 0.026667934 | 6.19398E-06 | 0.162945786 | TRUE |
| Diabetic kidney disease | Family Bacteroidales S24.7group | rs12505710 | 4 | 8565712 | A | G | 0.1449 | -0.1754 | -0.003977545 | 0.0385 | 0.028538442 | 5.29298E-06 | 0.889153825 | TRUE |
| Diabetic kidney disease | Family Bacteroidales S24.7group | rs2471672 | 15 | 36531651 | A | G | 0.7697 | -0.136 | -0.024512259 | 0.0306 | 0.020232117 | 8.9929E-06 | 0.22568398 | TRUE |
| Diabetic kidney disease | Family Bacteroidales S24.7group | rs2476601 | 1 | 114377568 | G | A | 0.8529 | -0.1765 | -0.038009193 | 0.0361 | 0.027515811 | 1.04201E-06 | 0.167168826 | TRUE |
| Diabetic kidney disease | Family Bacteroidales S24.7group | rs2852217 | 11 | 120647226 | T | C | 0.7149 | 0.1356 | 0.018959433 | 0.0286 | 0.018371627 | 2.13501E-06 | 0.302074313 | TRUE |
| Diabetic kidney disease | Family Bacteroidales S24.7group | rs34771920 | 3 | 29816198 | T | A | 0.0887 | 0.2179 | 0.004855883 | 0.0462 | 0.027695921 | 2.38002E-06 | 0.86082155 | TRUE |
| Diabetic kidney disease | Family Bacteroidales S24.7group | rs34872471 | 10 | 114754071 | C | T | 0.1963 | 0.1551 | 0.004974029 | 0.0326 | 0.017904009 | 1.93999E-06 | 0.781153209 | TRUE |
| Diabetic kidney disease | Family Bacteroidales S24.7group | rs3957146 | 6 | 32681530 | C | T | 0.1149 | 0.7266 | 0.021090244 | 0.0426 | 0.025726869 | 4.43404E-65 | 0.412344401 | TRUE |
| Diabetic kidney disease | Family Bacteroidales S24.7group | rs55694679 | 2 | 238873308 | T | C | 0.06447 | -0.2388 | -0.078605102 | 0.0538 | 0.039586127 | 8.96396E-06 | 0.047069646 | TRUE |
| Diabetic kidney disease | Family Bacteroidales S24.7group | rs76817227 | 3 | 68270049 | T | C | 0.02721 | 0.3551 | 0.038788876 | 0.0803 | 0.048628987 | 9.72098E-06 | 0.425074054 | TRUE |
| Diabetic kidney disease | Family Bacteroidales S24.7group | rs7742705 | 6 | 145001253 | A | G | 0.2213 | 0.1442 | -0.019136704 | 0.0312 | 0.018129937 | 3.72598E-06 | 0.2911827 | TRUE |
| Diabetic kidney disease | Family Bacteroidales S24.7group | rs78670164 | 12 | 12663634 | G | A | 0.01907 | 0.4202 | 0.008403367 | 0.0949 | 0.049567109 | 9.51196E-06 | 0.865375736 | TRUE |
| Diabetic kidney disease | Family Christensenellaceae | rs10489621 | 1 | 57238528 | C | T | 0.12 | 0.1743 | -0.014559061 | 0.0394 | 0.016566157 | 0.00000992 | 0.379486084 | TRUE |
| Diabetic kidney disease | Family Christensenellaceae | rs10507642 | 13 | 60481883 | C | T | 0.119 | 0.1801 | -0.002691552 | 0.0399 | 0.01857818 | 0.00000624 | 0.884807965 | TRUE |
| Diabetic kidney disease | Family Christensenellaceae | rs111366116 | 5 | 53295546 | T | C | 0.1207 | 0.1789 | -0.003820941 | 0.0396 | 0.018282285 | 0.00000619 | 0.834450646 | TRUE |
| Diabetic kidney disease | Family Christensenellaceae | rs12505710 | 4 | 8565712 | A | G | 0.1449 | -0.1754 | -0.027801754 | 0.0385 | 0.019763097 | 0.00000529 | 0.159501262 | TRUE |
| Diabetic kidney disease | Family Christensenellaceae | rs2471672 | 15 | 36531651 | A | G | 0.7697 | -0.136 | -0.010989254 | 0.0306 | 0.013977152 | 0.00000899 | 0.431732873 | TRUE |
| Diabetic kidney disease | Family Christensenellaceae | rs2476601 | 1 | 114377568 | G | A | 0.8529 | -0.1765 | -0.041237867 | 0.0361 | 0.019067566 | 0.00000104 | 0.030562478 | TRUE |
| Diabetic kidney disease | Family Christensenellaceae | rs2852217 | 11 | 120647226 | T | C | 0.7149 | 0.1356 | 0.003145566 | 0.0286 | 0.01263641 | 0.00000214 | 0.803415868 | TRUE |
| Diabetic kidney disease | Family Christensenellaceae | rs34771920 | 3 | 29816198 | T | A | 0.0887 | 0.2179 | 0.033382908 | 0.0462 | 0.018855236 | 0.00000238 | 0.076646427 | TRUE |
| Diabetic kidney disease | Family Christensenellaceae | rs34872471 | 10 | 114754071 | C | T | 0.1963 | 0.1551 | 0.00368501 | 0.0326 | 0.012232043 | 0.00000194 | 0.763217227 | TRUE |
| Diabetic kidney disease | Family Christensenellaceae | rs3957146 | 6 | 32681530 | C | T | 0.1149 | 0.7266 | 0.029855237 | 0.0426 | 0.017746109 | 4.43E-65 | 0.092500232 | TRUE |
| Diabetic kidney disease | Family Christensenellaceae | rs55694679 | 2 | 238873308 | T | C | 0.06447 | -0.2388 | 0.01849032 | 0.0538 | 0.028673657 | 0.00000896 | 0.519021853 | TRUE |
| Diabetic kidney disease | Family Christensenellaceae | rs76817227 | 3 | 68270049 | T | C | 0.02721 | 0.3551 | 0.022219135 | 0.0803 | 0.03065495 | 0.00000972 | 0.468566187 | TRUE |
| Diabetic kidney disease | Family Christensenellaceae | rs7742705 | 6 | 145001253 | A | G | 0.2213 | 0.1442 | 0.016986093 | 0.0312 | 0.012474425 | 0.00000373 | 0.173300961 | TRUE |
| Diabetic kidney disease | Family Christensenellaceae | rs78670164 | 12 | 12663634 | G | A | 0.01907 | 0.4202 | -0.051158143 | 0.0949 | 0.034970527 | 0.00000951 | 0.143496632 | TRUE |
| Diabetic kidney disease | Family Rhodospirillaceae | rs10489621 | 1 | 57238528 | C | T | 0.12 | 0.1743 | 0.02610502 | 0.0394 | 0.023918233 | 9.92202E-06 | 0.275084768 | TRUE |
| Diabetic kidney disease | Family Rhodospirillaceae | rs10507642 | 13 | 60481883 | C | T | 0.119 | 0.1801 | 0.020503311 | 0.0399 | 0.025772347 | 6.24195E-06 | 0.426290923 | TRUE |
| Diabetic kidney disease | Family Rhodospirillaceae | rs111366116 | 5 | 53295546 | T | C | 0.1207 | 0.1789 | 0.050697778 | 0.0396 | 0.025189845 | 6.19398E-06 | 0.04415382 | TRUE |
| Diabetic kidney disease | Family Rhodospirillaceae | rs12505710 | 4 | 8565712 | A | G | 0.1449 | -0.1754 | -0.044183341 | 0.0385 | 0.027496227 | 5.29298E-06 | 0.108079071 | TRUE |
| Diabetic kidney disease | Family Rhodospirillaceae | rs2471672 | 15 | 36531651 | A | G | 0.7697 | -0.136 | 0.004939907 | 0.0306 | 0.019987759 | 8.9929E-06 | 0.804794774 | TRUE |
| Diabetic kidney disease | Family Rhodospirillaceae | rs2476601 | 1 | 114377568 | G | A | 0.8529 | -0.1765 | -0.022691699 | 0.0361 | 0.026374413 | 1.04201E-06 | 0.389586261 | TRUE |
| Diabetic kidney disease | Family Rhodospirillaceae | rs2852217 | 11 | 120647226 | T | C | 0.7149 | 0.1356 | -0.000534329 | 0.0286 | 0.018108789 | 2.13501E-06 | 0.976460546 | TRUE |
| Diabetic kidney disease | Family Rhodospirillaceae | rs34771920 | 3 | 29816198 | T | A | 0.0887 | 0.2179 | 0.001107654 | 0.0462 | 0.026394787 | 2.38002E-06 | 0.966526711 | TRUE |
| Diabetic kidney disease | Family Rhodospirillaceae | rs34872471 | 10 | 114754071 | C | T | 0.1963 | 0.1551 | 0.017270627 | 0.0326 | 0.017108719 | 1.93999E-06 | 0.312752401 | TRUE |
| Diabetic kidney disease | Family Rhodospirillaceae | rs3957146 | 6 | 32681530 | C | T | 0.1149 | 0.7266 | 0.044059066 | 0.0426 | 0.025008305 | 4.43404E-65 | 0.078106921 | TRUE |
| Diabetic kidney disease | Family Rhodospirillaceae | rs55694679 | 2 | 238873308 | T | C | 0.06447 | -0.2388 | -0.061754398 | 0.0538 | 0.041397696 | 8.96396E-06 | 0.135768595 | TRUE |
| Diabetic kidney disease | Family Rhodospirillaceae | rs76817227 | 3 | 68270049 | T | C | 0.02721 | 0.3551 | -0.014553194 | 0.0803 | 0.043058377 | 9.72098E-06 | 0.735372587 | TRUE |
| Diabetic kidney disease | Family Rhodospirillaceae | rs7742705 | 6 | 145001253 | A | G | 0.2213 | 0.1442 | -0.003140606 | 0.0312 | 0.017685594 | 3.72598E-06 | 0.85905292 | TRUE |
| Diabetic kidney disease | Family Rhodospirillaceae | rs78670164 | 12 | 12663634 | G | A | 0.01907 | 0.4202 | -0.029614498 | 0.0949 | 0.046453315 | 9.51196E-06 | 0.523792072 | TRUE |
| Diabetic kidney disease | Genus Eubacteriumrectale | rs10489621 | 1 | 57238528 | C | T | 0.12 | 0.1743 | -0.002789156 | 0.0394 | 0.01624511 | 9.92202E-06 | 0.863679664 | TRUE |
| Diabetic kidney disease | Genus Eubacteriumrectale | rs10507642 | 13 | 60481883 | C | T | 0.119 | 0.1801 | 0.028062482 | 0.0399 | 0.018255209 | 6.24195E-06 | 0.12423664 | TRUE |
| Diabetic kidney disease | Genus Eubacteriumrectale | rs111366116 | 5 | 53295546 | T | C | 0.1207 | 0.1789 | -0.005254732 | 0.0396 | 0.018063752 | 6.19398E-06 | 0.771128375 | TRUE |
| Diabetic kidney disease | Genus Eubacteriumrectale | rs12505710 | 4 | 8565712 | A | G | 0.1449 | -0.1754 | 0.031897349 | 0.0385 | 0.019631082 | 5.29298E-06 | 0.104196851 | TRUE |
| Diabetic kidney disease | Genus Eubacteriumrectale | rs2471672 | 15 | 36531651 | A | G | 0.7697 | -0.136 | 0.008387117 | 0.0306 | 0.013718662 | 8.9929E-06 | 0.540957613 | TRUE |
| Diabetic kidney disease | Genus Eubacteriumrectale | rs2476601 | 1 | 114377568 | G | A | 0.8529 | -0.1765 | 0.015545734 | 0.0361 | 0.018934032 | 1.04201E-06 | 0.411619386 | TRUE |
| Diabetic kidney disease | Genus Eubacteriumrectale | rs2852217 | 11 | 120647226 | T | C | 0.7149 | 0.1356 | 0.001467285 | 0.0286 | 0.012448692 | 2.13501E-06 | 0.906173372 | TRUE |
| Diabetic kidney disease | Genus Eubacteriumrectale | rs34771920 | 3 | 29816198 | T | A | 0.0887 | 0.2179 | -0.018547303 | 0.0462 | 0.018581007 | 2.38002E-06 | 0.318189128 | TRUE |
| Diabetic kidney disease | Genus Eubacteriumrectale | rs34872471 | 10 | 114754071 | C | T | 0.1963 | 0.1551 | -0.024383246 | 0.0326 | 0.012050508 | 1.93999E-06 | 0.0430298 | TRUE |
| Diabetic kidney disease | Genus Eubacteriumrectale | rs3957146 | 6 | 32681530 | C | T | 0.1149 | 0.7266 | -0.035702965 | 0.0426 | 0.017453508 | 4.43404E-65 | 0.040795351 | TRUE |
| Diabetic kidney disease | Genus Eubacteriumrectale | rs55694679 | 2 | 238873308 | T | C | 0.06447 | -0.2388 | -0.029471438 | 0.0538 | 0.027972632 | 8.96396E-06 | 0.292074713 | TRUE |
| Diabetic kidney disease | Genus Eubacteriumrectale | rs76817227 | 3 | 68270049 | T | C | 0.02721 | 0.3551 | -0.071030777 | 0.0803 | 0.030244961 | 9.72098E-06 | 0.018848387 | TRUE |
| Diabetic kidney disease | Genus Eubacteriumrectale | rs7742705 | 6 | 145001253 | A | G | 0.2213 | 0.1442 | -0.010987469 | 0.0312 | 0.012268075 | 3.72598E-06 | 0.370458585 | TRUE |
| Diabetic kidney disease | Genus Eubacteriumrectale | rs78670164 | 12 | 12663634 | G | A | 0.01907 | 0.4202 | -0.007414878 | 0.0949 | 0.03469998 | 9.51196E-06 | 0.830792423 | TRUE |
| Diabetic kidney disease | Genus Adlercreutzia | rs10489621 | 1 | 57238528 | C | T | 0.12 | 0.1743 | 0.006277228 | 0.0394 | 0.024094004 | 9.92202E-06 | 0.794454433 | TRUE |
| Diabetic kidney disease | Genus Adlercreutzia | rs10507642 | 13 | 60481883 | C | T | 0.119 | 0.1801 | 0.026436576 | 0.0399 | 0.026969009 | 6.24195E-06 | 0.326958969 | TRUE |
| Diabetic kidney disease | Genus Adlercreutzia | rs111366116 | 5 | 53295546 | T | C | 0.1207 | 0.1789 | 0.038422871 | 0.0396 | 0.02562957 | 6.19398E-06 | 0.133831671 | TRUE |
| Diabetic kidney disease | Genus Adlercreutzia | rs12505710 | 4 | 8565712 | A | G | 0.1449 | -0.1754 | -0.010932076 | 0.0385 | 0.027214947 | 5.29298E-06 | 0.687909367 | TRUE |
| Diabetic kidney disease | Genus Adlercreutzia | rs2471672 | 15 | 36531651 | A | G | 0.7697 | -0.136 | -0.00421283 | 0.0306 | 0.020434335 | 8.9929E-06 | 0.836662603 | TRUE |
| Diabetic kidney disease | Genus Adlercreutzia | rs2476601 | 1 | 114377568 | G | A | 0.8529 | -0.1765 | -0.017481813 | 0.0361 | 0.026118267 | 1.04201E-06 | 0.503283187 | TRUE |
| Diabetic kidney disease | Genus Adlercreutzia | rs2852217 | 11 | 120647226 | T | C | 0.7149 | 0.1356 | -0.014027818 | 0.0286 | 0.017842698 | 2.13501E-06 | 0.431753949 | TRUE |
| Diabetic kidney disease | Genus Adlercreutzia | rs34771920 | 3 | 29816198 | T | A | 0.0887 | 0.2179 | 0.004364938 | 0.0462 | 0.026135183 | 2.38002E-06 | 0.867359148 | TRUE |
| Diabetic kidney disease | Genus Adlercreutzia | rs34872471 | 10 | 114754071 | C | T | 0.1963 | 0.1551 | 0.019527646 | 0.0326 | 0.017370801 | 1.93999E-06 | 0.260943042 | TRUE |
| Diabetic kidney disease | Genus Adlercreutzia | rs3957146 | 6 | 32681530 | C | T | 0.1149 | 0.7266 | 0.007527207 | 0.0426 | 0.026101085 | 4.43404E-65 | 0.773050719 | TRUE |
| Diabetic kidney disease | Genus Adlercreutzia | rs55694679 | 2 | 238873308 | T | C | 0.06447 | -0.2388 | 0.016001909 | 0.0538 | 0.044132378 | 8.96396E-06 | 0.716912028 | TRUE |
| Diabetic kidney disease | Genus Adlercreutzia | rs76817227 | 3 | 68270049 | T | C | 0.02721 | 0.3551 | 0.033352331 | 0.0803 | 0.044157497 | 9.72098E-06 | 0.450066642 | TRUE |
| Diabetic kidney disease | Genus Adlercreutzia | rs7742705 | 6 | 145001253 | A | G | 0.2213 | 0.1442 | 0.022019487 | 0.0312 | 0.018050859 | 3.72598E-06 | 0.222518677 | TRUE |
| Diabetic kidney disease | Genus Adlercreutzia | rs78670164 | 12 | 12663634 | G | A | 0.01907 | 0.4202 | 0.117349831 | 0.0949 | 0.048316634 | 9.51196E-06 | 0.015150278 | TRUE |
| Diabetic kidney disease | Genus FamilyXIIIUCG001 | rs10489621 | 1 | 57238528 | C | T | 0.12 | 0.1743 | -0.017526 | 0.0394 | 0.019314853 | 9.92202E-06 | 0.364203464 | TRUE |
| Diabetic kidney disease | Genus FamilyXIIIUCG001 | rs10507642 | 13 | 60481883 | C | T | 0.119 | 0.1801 | -0.045781145 | 0.0399 | 0.021809638 | 6.24195E-06 | 0.035805927 | TRUE |
| Diabetic kidney disease | Genus FamilyXIIIUCG001 | rs111366116 | 5 | 53295546 | T | C | 0.1207 | 0.1789 | 0.007621805 | 0.0396 | 0.020817969 | 6.19398E-06 | 0.714278038 | TRUE |
| Diabetic kidney disease | Genus FamilyXIIIUCG001 | rs12505710 | 4 | 8565712 | A | G | 0.1449 | -0.1754 | -0.035689996 | 0.0385 | 0.021955115 | 5.29298E-06 | 0.104037093 | TRUE |
| Diabetic kidney disease | Genus FamilyXIIIUCG001 | rs2471672 | 15 | 36531651 | A | G | 0.7697 | -0.136 | 0.013266327 | 0.0306 | 0.016342368 | 8.9929E-06 | 0.416920704 | TRUE |
| Diabetic kidney disease | Genus FamilyXIIIUCG001 | rs2476601 | 1 | 114377568 | G | A | 0.8529 | -0.1765 | -0.019926875 | 0.0361 | 0.02121288 | 1.04201E-06 | 0.347537607 | TRUE |
| Diabetic kidney disease | Genus FamilyXIIIUCG001 | rs2852217 | 11 | 120647226 | T | C | 0.7149 | 0.1356 | 0.006196302 | 0.0286 | 0.014464255 | 2.13501E-06 | 0.668369226 | TRUE |
| Diabetic kidney disease | Genus FamilyXIIIUCG001 | rs34771920 | 3 | 29816198 | T | A | 0.0887 | 0.2179 | -0.033139085 | 0.0462 | 0.021566895 | 2.38002E-06 | 0.124398171 | TRUE |
| Diabetic kidney disease | Genus FamilyXIIIUCG001 | rs34872471 | 10 | 114754071 | C | T | 0.1963 | 0.1551 | -0.033449172 | 0.0326 | 0.014044268 | 1.93999E-06 | 0.017233135 | TRUE |
| Diabetic kidney disease | Genus FamilyXIIIUCG001 | rs3957146 | 6 | 32681530 | C | T | 0.1149 | 0.7266 | -0.036640149 | 0.0426 | 0.020747009 | 4.43404E-65 | 0.077388313 | TRUE |
| Diabetic kidney disease | Genus FamilyXIIIUCG001 | rs55694679 | 2 | 238873308 | T | C | 0.06447 | -0.2388 | -0.01767205 | 0.0538 | 0.034072269 | 8.96396E-06 | 0.603995264 | TRUE |
| Diabetic kidney disease | Genus FamilyXIIIUCG001 | rs76817227 | 3 | 68270049 | T | C | 0.02721 | 0.3551 | -0.058339284 | 0.0803 | 0.036113225 | 9.72098E-06 | 0.106212233 | TRUE |
| Diabetic kidney disease | Genus FamilyXIIIUCG001 | rs7742705 | 6 | 145001253 | A | G | 0.2213 | 0.1442 | -0.003037171 | 0.0312 | 0.014474452 | 3.72598E-06 | 0.83380052 | TRUE |
| Diabetic kidney disease | Genus FamilyXIIIUCG001 | rs78670164 | 12 | 12663634 | G | A | 0.01907 | 0.4202 | -0.020771391 | 0.0949 | 0.038838112 | 9.51196E-06 | 0.592774478 | TRUE |
| Diabetic kidney disease | Genus LachnospiraceaeNC2004 | rs10489621 | 1 | 57238528 | C | T | 0.12 | 0.1743 | 0.040799388 | 0.0394 | 0.025763268 | 9.92202E-06 | 0.113278797 | TRUE |
| Diabetic kidney disease | Genus LachnospiraceaeNC2004 | rs10507642 | 13 | 60481883 | C | T | 0.119 | 0.1801 | -0.005769192 | 0.0399 | 0.032617255 | 6.24195E-06 | 0.859606223 | TRUE |
| Diabetic kidney disease | Genus LachnospiraceaeNC2004 | rs111366116 | 5 | 53295546 | T | C | 0.1207 | 0.1789 | 0.042710294 | 0.0396 | 0.03096481 | 6.19398E-06 | 0.167796997 | TRUE |
| Diabetic kidney disease | Genus LachnospiraceaeNC2004 | rs12505710 | 4 | 8565712 | A | G | 0.1449 | -0.1754 | -0.028530142 | 0.0385 | 0.035244782 | 5.29298E-06 | 0.418235912 | TRUE |
| Diabetic kidney disease | Genus LachnospiraceaeNC2004 | rs2471672 | 15 | 36531651 | A | G | 0.7697 | -0.136 | 0.024392798 | 0.0306 | 0.022396441 | 8.9929E-06 | 0.276093378 | TRUE |
| Diabetic kidney disease | Genus LachnospiraceaeNC2004 | rs2476601 | 1 | 114377568 | G | A | 0.8529 | -0.1765 | 0.007412924 | 0.0361 | 0.032375048 | 1.04201E-06 | 0.818892017 | TRUE |
| Diabetic kidney disease | Genus LachnospiraceaeNC2004 | rs2852217 | 11 | 120647226 | T | C | 0.7149 | 0.1356 | -0.037256498 | 0.0286 | 0.020366909 | 2.13501E-06 | 0.067359732 | TRUE |
| Diabetic kidney disease | Genus LachnospiraceaeNC2004 | rs34771920 | 3 | 29816198 | T | A | 0.0887 | 0.2179 | 0.033419225 | 0.0462 | 0.031919629 | 2.38002E-06 | 0.295108638 | TRUE |
| Diabetic kidney disease | Genus LachnospiraceaeNC2004 | rs34872471 | 10 | 114754071 | C | T | 0.1963 | 0.1551 | 0.008947757 | 0.0326 | 0.019970003 | 1.93999E-06 | 0.654109993 | TRUE |
| Diabetic kidney disease | Genus LachnospiraceaeNC2004 | rs3957146 | 6 | 32681530 | C | T | 0.1149 | 0.7266 | 0.050990157 | 0.0426 | 0.030390412 | 4.43404E-65 | 0.093378928 | TRUE |
| Diabetic kidney disease | Genus LachnospiraceaeNC2004 | rs55694679 | 2 | 238873308 | T | C | 0.06447 | -0.2388 | 0.008828452 | 0.0538 | 0.040344658 | 8.96396E-06 | 0.826785743 | TRUE |
| Diabetic kidney disease | Genus LachnospiraceaeNC2004 | rs76817227 | 3 | 68270049 | T | C | 0.02721 | 0.3551 | 0.102974996 | 0.0803 | 0.05120978 | 9.72098E-06 | 0.044341698 | TRUE |
| Diabetic kidney disease | Genus LachnospiraceaeNC2004 | rs7742705 | 6 | 145001253 | A | G | 0.2213 | 0.1442 | 0.021614686 | 0.0312 | 0.020126299 | 3.72598E-06 | 0.282844045 | TRUE |
| Diabetic kidney disease | Genus LachnospiraceaeNC2004 | rs78670164 | 12 | 12663634 | G | A | 0.01907 | 0.4202 | -0.002643072 | 0.0949 | 0.052857025 | 9.51196E-06 | 0.960119063 | TRUE |
| Diabetic kidney disease | Genus LachnospiraceaeNC2004 | rs10489621 | 1 | 57238528 | C | T | 0.12 | 0.1743 | 0.011640755 | 0.0394 | 0.018709332 | 9.92202E-06 | 0.533817073 | TRUE |
| Diabetic kidney disease | Genus LachnospiraceaeNC2004 | rs10507642 | 13 | 60481883 | C | T | 0.119 | 0.1801 | 0.024875042 | 0.0399 | 0.021451937 | 6.24195E-06 | 0.246223557 | TRUE |
| Diabetic kidney disease | Genus LachnospiraceaeNC2004 | rs111366116 | 5 | 53295546 | T | C | 0.1207 | 0.1789 | 0.013473573 | 0.0396 | 0.020743929 | 6.19398E-06 | 0.516003059 | TRUE |
| Diabetic kidney disease | Genus LachnospiraceaeNC2004 | rs12505710 | 4 | 8565712 | A | G | 0.1449 | -0.1754 | -0.005482376 | 0.0385 | 0.022526744 | 5.29298E-06 | 0.807717328 | TRUE |
| Diabetic kidney disease | Genus LachnospiraceaeNC2004 | rs2471672 | 15 | 36531651 | A | G | 0.7697 | -0.136 | -0.0041099 | 0.0306 | 0.015793947 | 8.9929E-06 | 0.794694135 | TRUE |
| Diabetic kidney disease | Genus LachnospiraceaeNC2004 | rs2476601 | 1 | 114377568 | G | A | 0.8529 | -0.1765 | 0.012320713 | 0.0361 | 0.021560929 | 1.04201E-06 | 0.56770346 | TRUE |
| Diabetic kidney disease | Genus LachnospiraceaeNC2004 | rs2852217 | 11 | 120647226 | T | C | 0.7149 | 0.1356 | 0.018974596 | 0.0286 | 0.014290162 | 2.13501E-06 | 0.184241421 | TRUE |
| Diabetic kidney disease | Genus LachnospiraceaeNC2004 | rs34771920 | 3 | 29816198 | T | A | 0.0887 | 0.2179 | 0.011828667 | 0.0462 | 0.02158494 | 2.38002E-06 | 0.583688084 | TRUE |
| Diabetic kidney disease | Genus LachnospiraceaeNC2004 | rs34872471 | 10 | 114754071 | C | T | 0.1963 | 0.1551 | 0.002667492 | 0.0326 | 0.013795047 | 1.93999E-06 | 0.846672378 | TRUE |
| Diabetic kidney disease | Genus LachnospiraceaeNC2004 | rs3957146 | 6 | 32681530 | C | T | 0.1149 | 0.7266 | 0.037574237 | 0.0426 | 0.020280492 | 4.43404E-65 | 0.063921341 | TRUE |
| Diabetic kidney disease | Genus LachnospiraceaeNC2004 | rs55694679 | 2 | 238873308 | T | C | 0.06447 | -0.2388 | -0.04529233 | 0.0538 | 0.0313893 | 8.96396E-06 | 0.149042277 | TRUE |
| Diabetic kidney disease | Genus LachnospiraceaeNC2004 | rs76817227 | 3 | 68270049 | T | C | 0.02721 | 0.3551 | 0.038574014 | 0.0803 | 0.035515843 | 9.72098E-06 | 0.277431545 | TRUE |
| Diabetic kidney disease | Genus LachnospiraceaeNC2004 | rs7742705 | 6 | 145001253 | A | G | 0.2213 | 0.1442 | 0.007479668 | 0.0312 | 0.01406358 | 3.72598E-06 | 0.594832213 | TRUE |
| Diabetic kidney disease | Genus LachnospiraceaeNC2004 | rs78670164 | 12 | 12663634 | G | A | 0.01907 | 0.4202 | -0.04856575 | 0.0949 | 0.038950199 | 9.51196E-06 | 0.212445959 | TRUE |
| Diabetic kidney disease | Genus Turicibacter | rs10489621 | 1 | 57238528 | C | T | 0.12 | 0.1743 | 0.027854725 | 0.0394 | 0.022943204 | 9.92202E-06 | 0.224719831 | TRUE |
| Diabetic kidney disease | Genus Turicibacter | rs10507642 | 13 | 60481883 | C | T | 0.119 | 0.1801 | 0.012098744 | 0.0399 | 0.02566012 | 6.24195E-06 | 0.637283786 | TRUE |
| Diabetic kidney disease | Genus Turicibacter | rs111366116 | 5 | 53295546 | T | C | 0.1207 | 0.1789 | -0.005280095 | 0.0396 | 0.024836577 | 6.19398E-06 | 0.831644029 | TRUE |
| Diabetic kidney disease | Genus Turicibacter | rs12505710 | 4 | 8565712 | A | G | 0.1449 | -0.1754 | -0.004381201 | 0.0385 | 0.02626854 | 5.29298E-06 | 0.867539141 | TRUE |
| Diabetic kidney disease | Genus Turicibacter | rs2471672 | 15 | 36531651 | A | G | 0.7697 | -0.136 | -0.018669364 | 0.0306 | 0.019436592 | 8.9929E-06 | 0.33679024 | TRUE |
| Diabetic kidney disease | Genus Turicibacter | rs2476601 | 1 | 114377568 | G | A | 0.8529 | -0.1765 | 0.000426941 | 0.0361 | 0.025641473 | 1.04201E-06 | 0.986715501 | TRUE |
| Diabetic kidney disease | Genus Turicibacter | rs2852217 | 11 | 120647226 | T | C | 0.7149 | 0.1356 | -0.015241541 | 0.0286 | 0.017288878 | 2.13501E-06 | 0.378003599 | TRUE |
| Diabetic kidney disease | Genus Turicibacter | rs34771920 | 3 | 29816198 | T | A | 0.0887 | 0.2179 | 0.041653431 | 0.0462 | 0.025671921 | 2.38002E-06 | 0.10469017 | TRUE |
| Diabetic kidney disease | Genus Turicibacter | rs34872471 | 10 | 114754071 | C | T | 0.1963 | 0.1551 | -0.023347379 | 0.0326 | 0.016778393 | 1.93999E-06 | 0.164069427 | TRUE |
| Diabetic kidney disease | Genus Turicibacter | rs3957146 | 6 | 32681530 | C | T | 0.1149 | 0.7266 | 0.064217054 | 0.0426 | 0.024444589 | 4.43404E-65 | 0.008612974 | TRUE |
| Diabetic kidney disease | Genus Turicibacter | rs55694679 | 2 | 238873308 | T | C | 0.06447 | -0.2388 | -0.009520898 | 0.0538 | 0.041473362 | 8.96396E-06 | 0.818428583 | TRUE |
| Diabetic kidney disease | Genus Turicibacter | rs76817227 | 3 | 68270049 | T | C | 0.02721 | 0.3551 | -0.040057224 | 0.0803 | 0.04125652 | 9.72098E-06 | 0.331582761 | TRUE |
| Diabetic kidney disease | Genus Turicibacter | rs7742705 | 6 | 145001253 | A | G | 0.2213 | 0.1442 | -0.00742319 | 0.0312 | 0.017210873 | 3.72598E-06 | 0.666244308 | TRUE |
| Diabetic kidney disease | Genus Turicibacter | rs78670164 | 12 | 12663634 | G | A | 0.01907 | 0.4202 | -0.000132225 | 0.0949 | 0.048832065 | 9.51196E-06 | 0.997839532 | TRUE |

**Table S7 The reverse MR results of causal effects of diabetic kidney disease on gut microbiota.**

|  |  |  |  |  |  |  |  |  |  |  | **c** | | | **Heterogeneity （Cochran's Q test)** | | **MR-PRESSO** |  |
| --- | --- | --- | --- | --- | --- | --- | --- | --- | --- | --- | --- | --- | --- | --- | --- | --- | --- |
| **Outcomecccc** | **Method** | **Nsnp** | **b** | **se** | **OR** | **95%CI_low** | **95%CI_up** | **P value** | **P**_adjust_ | **Directionccccc** | **Egger intercept** | **SE** | **P value** | **Q** | **P value** | **P value** | |
| Phylum Actinobacteria |  |  |  |  |  |  |  |  |  |  |  |  |  |  |  |  | |
|  | MR Egger | 14 | -0.000526 | 0.030094 | 0.999474 | 0.94222509 | 1.060201368 | 0.986339 | 0.986339 |  |  |  |  |  |  |  | |
|  | Weighted median | 14 | 0.0155385 | 0.021737 | 0.984582 | 0.94351417 | 1.027436526 | 0.484009 | 0.693618 |  |  |  |  |  |  |  | |
|  | Inverse variance weight | 14 | -0.039289 | 0.017302 | 0.961473 | 0.92941393 | 0.99463797 | 0.023163 | 0.104233 | decreased | -0.0120 | 0.0076 | 0.1414 | 12.25 | 0.5069 | 0.4560 | |
|  | Simple mode | 14 | 0.0193644 | 0.041151 | 0.980822 | 0.904818902 | 1.063208878 | 0.638183 | 0.82098 |  |  |  |  |  |  |  | |
|  | Weighted mode | 14 | 0.0142246 | 0.023036 | 0.985876 | 0.942352402 | 1.031410046 | 0.52295 | 0.724594 |  |  |  |  |  |  |  | |
| Phylum Proteobacteria |  |  |  |  |  |  |  |  |  |  |  |  |  |  |  |  | |
|  | MR Egger | 14 | 0.0426347 | 0.03046 | 1.043557 | 0.98307879 | 1.107755015 | 0.186916 | 0.829523 |  |  |  |  |  |  |  | |
|  | Weighted median | 14 | 0.0498689 | 0.02194 | 1.051133 | 1.006889691 | 1.097321002 | 0.018582 | 0.167234 |  |  |  |  |  |  |  | |
|  | Inverse variance weight | 14 | 0.0498223 | 0.017161 | 1.051084 | 1.016318497 | 1.087039327 | 0.003693 | 0.03324 | increased | 0.0022 | 0.0078 | 0.7778 | 12.62 | 0.4775 | 0.5903 | |
|  | Simple mode | 14 | 0.0486522 | 0.042318 | 1.049855 | 0.966291097 | 1.140645722 | 0.256096 | 0.82098 |  |  |  |  |  |  |  | |
|  | Weighted mode | 14 | 0.0497528 | 0.024023 | 1.051011 | 1.002670896 | 1.10168217 | 0.049133 | 0.332703 |  |  |  |  |  |  |  | |
| Class Alphaproteobacteria |  |  |  |  |  |  |  |  |  |  |  |  |  |  |  |  | |
|  | MR Egger | 14 | 0.0243781 | 0.041158 | 1.024678 | 0.945263194 | 1.11076398 | 0.564642 | 0.999822 |  |  |  |  |  |  |  | |
|  | Weighted median | 14 | 0.0582431 | 0.031055 | 1.059973 | 0.997377879 | 1.126495857 | 0.0668 | 0.488794 |  |  |  |  |  |  |  | |
|  | Inverse variance weight | 14 | 0.069131 | 0.023656 | 1.071577 | 1.023025952 | 1.122431291 | 0.003474 | 0.055589 | increased | 0.0139 | 0.0106 | 0.2086 | 12.68 | 0.4730 | 0.5443 | |
|  | Simple mode | 14 | 0.1027433 | 0.063837 | 1.108207 | 0.977871854 | 1.25591344 | 0.128216 | 0.677975 |  |  |  |  |  |  |  | |
|  | Weighted mode | 14 | 0.0558971 | 0.031949 | 1.057489 | 0.993300386 | 1.125825397 | 0.102796 | 0.652032 |  |  |  |  |  |  |  | |
| Class Betaproteobacteria |  |  |  |  |  |  |  |  |  |  |  |  |  |  |  |  | |
|  | MR Egger | 14 | 0.0138155 | 0.033834 | 1.013911 | 0.948855561 | 1.083427576 | 0.69022 | 0.999822 |  |  |  |  |  |  |  | |
|  | Weighted median | 14 | 0.0298422 | 0.021817 | 1.030292 | 0.987164305 | 1.075303686 | 0.183266 | 0.488794 |  |  |  |  |  |  |  | |
|  | Inverse variance weight | 14 | 0.0380231 | 0.019298 | 1.038755 | 1.000199964 | 1.07879668 | 0.048797 | 0.390376 | increased | 0.0075 | 0.0086 | 0.3988 | 15.44 | 0.2809 | 0.3473 | |
|  | Simple mode | 14 | 0.1011756 | 0.051266 | 1.106471 | 1.000694125 | 1.223428635 | 0.071339 | 0.677975 |  |  |  |  |  |  |  | |
|  | Weighted mode | 14 | 0.027132 | 0.023414 | 1.027503 | 0.981416202 | 1.075754847 | 0.262521 | 0.652032 |  |  |  |  |  |  |  | |
| Order Rhodospirillales |  |  |  |  |  |  |  |  |  |  |  |  |  |  |  |  | |
|  | MR Egger | 14 | 0.0451098 | 0.043512 | 1.046143 | 0.96062281 | 1.139276061 | 0.320305 | 0.837266 |  |  |  |  |  |  |  | |
|  | Weighted median | 14 | 0.0559817 | 0.032454 | 1.057578 | 0.992401385 | 1.127035738 | 0.087348 | 0.640231 |  |  |  |  |  |  |  | |
|  | Inverse variance weight | 14 | 0.0626175 | 0.024991 | 1.06462 | 1.013727988 | 1.118066037 | 0.012225 | 0.244505 | increased | 0.0055 | 0.0111 | 0.6319 | 10.80 | 0.6277 | 0.7204 | |
|  | Simple mode | 14 | 0.0207619 | 0.065561 | 0.979452 | 0.861344071 | 1.113755269 | 0.743147 | 0.795318 |  |  |  |  |  |  |  | |
|  | Weighted mode | 14 | 0.054424 | 0.033477 | 1.055932 | 0.988871408 | 1.127540746 | 0.147333 | 0.665669 |  |  |  |  |  |  |  | |
| Family Bacteroidales S24.7group |  |  |  |  |  |  |  |  |  |  |  |  |  |  |  |  | |
|  | MR Egger | 14 | 0.0217615 | 0.045143 | 1.022 | 0.935457857 | 1.116548399 | 0.63844 | 0.941092 |  |  |  |  |  |  |  | |
|  | Weighted median | 14 | 0.0293254 | 0.031637 | 1.02976 | 0.96784504 | 1.095634913 | 0.361243 | 0.679987 |  |  |  |  |  |  |  | |
|  | Inverse variance weight | 14 | 0.057261 | 0.025992 | 1.058932 | 1.006337667 | 1.114275511 | 0.02759 | 0.354503 | increased | 0.0110 | 0.0115 | 0.3551 | 10.97 | 0.6136 | 0.6077 | |
|  | Simple mode | 14 | 0.0291159 | 0.065866 | 1.029544 | 0.904854741 | 1.171415247 | 0.686937 | 0.980603 |  |  |  |  |  |  |  | |
|  | Weighted mode | 14 | 0.0291159 | 0.034243 | 1.029544 | 0.962712589 | 1.101014624 | 0.419285 | 0.745395 |  |  |  |  |  |  |  | |
| Family Christensenellaceae |  |  |  |  |  |  |  |  |  |  |  |  |  |  |  |  | |
|  | MR Egger | 14 | 0.0258679 | 0.033483 | 1.026205 | 0.961021353 | 1.095810766 | 0.454725 | 0.941092 |  |  |  |  |  |  |  | |
|  | Weighted median | 14 | 0.0395378 | 0.023099 | 1.04033 | 0.994279269 | 1.088513297 | 0.091904 | 0.594598 |  |  |  |  |  |  |  | |
|  | Inverse variance weight | 14 | 0.0398082 | 0.018696 | 1.040611 | 1.003169055 | 1.079450688 | 0.033235 | 0.354503 | increased | 0.0043 | 0.0085 | 0.6201 | 14.21 | 0.3593 | 0.4666 | |
|  | Simple mode | 14 | 0.0313245 | 0.050545 | 1.03182 | 0.934499191 | 1.139276645 | 0.537365 | 0.980603 |  |  |  |  |  |  |  | |
|  | Weighted mode | 14 | 0.0406414 | 0.022478 | 1.041479 | 0.996590631 | 1.088388212 | 0.119891 | 0.714543 |  |  |  |  |  |  |  | |
| Family Rhodospirillaceae |  |  |  |  |  |  |  |  |  |  |  |  |  |  |  |  | |
|  | MR Egger | 14 | 0.0463288 | 0.043764 | 1.047419 | 0.961318531 | 1.141230512 | 0.310628 | 0.941092 |  |  |  |  |  |  |  | |
|  | Weighted median | 14 | 0.0571412 | 0.032024 | 1.058805 | 0.994390163 | 1.127393052 | 0.07426 | 0.594598 |  |  |  |  |  |  |  | |
|  | Inverse variance weight | 14 | 0.0638769 | 0.025124 | 1.065961 | 1.014742379 | 1.119765184 | 0.011006 | 0.352188 | increased | 0.0055 | 0.0112 | 0.6332 | 9.69 | 0.7185 | 0.7947 | |
|  | Simple mode | 14 | 0.0035671 | 0.063266 | 0.996439 | 0.88023373 | 1.127985836 | 0.955012 | 0.980603 |  |  |  |  |  |  |  | |
|  | Weighted mode | 14 | 0.0568369 | 0.032849 | 1.058483 | 0.992482015 | 1.128873465 | 0.112341 | 0.714543 |  |  |  |  |  |  |  | |
| Genus *Eubacterium rectale* group |  |  |  |  |  |  |  |  |  |  |  |  |  |  |  |  | |
|  | MR Egger | 14 | 0.0508666 | 0.032869 | 0.950405 | 0.891107732 | 1.013649031 | 0.147686 | 0.919549 |  |  |  |  |  |  |  | |
|  | Weighted median | 14 | 0.0500443 | 0.022376 | 0.951187 | 0.910372381 | 0.993832025 | 0.025424 | 0.743065 |  |  |  |  |  |  |  | |
|  | Inverse variance weight | 14 | 0.0530402 | 0.018174 | 0.948342 | 0.91515553 | 0.982731684 | 0.003518 | 0.418593 | decreased | -0.0007 | 0.0084 | 0.9369 | 13.85 | 0.3843 | 0.5076 | |
|  | Simple mode | 14 | 0.0521156 | 0.044988 | 0.949219 | 0.869104381 | 1.036718974 | 0.260485 | 0.991813 |  |  |  |  |  |  |  | |
|  | Weighted mode | 14 | 0.0489568 | 0.023433 | 0.952222 | 0.909476841 | 0.996976743 | 0.044755 | 0.797147 |  |  |  |  |  |  |  | |
| Genus *Adlercreutzia* |  |  |  |  |  |  |  |  |  |  |  |  |  |  |  |  | |
|  | MR Egger | 14 | 0.0200128 | 0.045475 | 1.020214 | 0.933215968 | 1.115323299 | 0.6677 | 0.944867 |  |  |  |  |  |  |  | |
|  | Weighted median | 14 | 0.0161933 | 0.03356 | 1.016325 | 0.951625604 | 1.085423541 | 0.625425 | 0.89176 |  |  |  |  |  |  |  | |
|  | Inverse variance weight | 14 | 0.0508157 | 0.025885 | 1.052129 | 1.000081562 | 1.106884993 | 0.049629 | 0.707129 | increased | 0.0094 | 0.0114 | 0.4261 | 10.16 | 0.6811 | 0.5758 | |
|  | Simple mode | 14 | 0.0672869 | 0.069915 | 1.069602 | 0.93263064 | 1.226690448 | 0.360504 | 0.991813 |  |  |  |  |  |  |  | |
|  | Weighted mode | 14 | 0.0180559 | 0.032782 | 1.01822 | 0.954853302 | 1.085791721 | 0.594328 | 0.896369 |  |  |  |  |  |  |  | |
| Genus FamilyXIIIUCG001 |  |  |  |  |  |  |  |  |  |  |  |  |  |  |  |  | |
|  | MR Egger | 14 | 0.0564934 | 0.043179 | 0.945073 | 0.868381367 | 1.028537113 | 0.21526 | 0.919549 |  |  |  |  |  |  |  | |
|  | Weighted median | 14 | 0.0503741 | 0.025512 | 0.950874 | 0.904495433 | 0.99962987 | 0.059097 | 0.743065 |  |  |  |  |  |  |  | |
|  | Inverse variance weight | 14 | 0.0550718 | 0.023735 | 0.946417 | 0.903398317 | 0.991484663 | 0.020324 | 0.707129 | decreased | 0.0004 | 0.0109 | 0.9686 | 17.04 | 0.1976 | 0.3052 | |
|  | Simple mode | 14 | 0.0655425 | 0.05886 | 0.936559 | 0.834512815 | 1.051084187 | 0.291161 | 0.991813 |  |  |  |  |  |  |  | |
|  | Weighted mode | 14 | 0.0518635 | 0.026945 | 0.949458 | 0.90061669 | 1.000949046 | 0.064724 | 0.797147 |  |  |  |  |  |  |  | |
| Genus LachnospiraceaeNC2004 |  |  |  |  |  |  |  |  |  |  |  |  |  |  |  |  | |
|  | MR Egger | 14 | 0.101075 | 0.056129 | 1.10636 | 0.99110186 | 1.235020923 | 0.096907 | 0.919549 |  |  |  |  |  |  |  | |
|  | Weighted median | 14 | 0.0689133 | 0.03679 | 1.071343 | 0.996809054 | 1.151450663 | 0.070237 | 0.743065 |  |  |  |  |  |  |  | |
|  | Inverse variance weight | 14 | 0.0645381 | 0.031394 | 1.066666 | 1.003010421 | 1.134361973 | 0.039807 | 0.707129 | increased | -0.0111 | 0.0139 | 0.4445 | 14.37 | 0.3483 | 0.4496 | |
|  | Simple mode | 14 | 0.0329636 | 0.075015 | 1.033513 | 0.892200117 | 1.197207925 | 0.686785 | 0.991813 |  |  |  |  |  |  |  | |
|  | Weighted mode | 14 | 0.0674339 | 0.039437 | 1.06976 | 0.990185419 | 1.155728407 | 0.086181 | 0.797147 |  |  |  |  |  |  |  | |
| Genus LachnospiraceaeUCG010 |  |  |  |  |  |  |  |  |  |  |  |  |  |  |  |  | |
|  | MR Egger | 14 | 0.0414809 | 0.035472 | 1.042353 | 0.972345439 | 1.117401651 | 0.264946 | 0.919549 |  |  |  |  |  |  |  | |
|  | Weighted median | 14 | 0.0517382 | 0.025112 | 1.0531 | 1.002521937 | 1.106229672 | 0.054991 | 0.743065 |  |  |  |  |  |  |  | |
|  | Inverse variance weight | 14 | 0.0501555 | 0.02035 | 1.051435 | 1.010322606 | 1.094219398 | 0.013714 | 0.707129 | increased | 0.0027 | 0.0089 | 0.7704 | 7.17 | 0.8934 | 0.9259 | |
|  | Simple mode | 14 | 0.0487727 | 0.043597 | 1.049982 | 0.963987139 | 1.143647624 | 0.340347 | 0.991813 |  |  |  |  |  |  |  | |
|  | Weighted mode | 14 | 0.0513791 | 0.026691 | 1.052722 | 0.999064637 | 1.10926089 | 0.082689 | 0.797147 |  |  |  |  |  |  |  | |
| Genus Turicibacter |  |  |  |  |  |  |  |  |  |  |  |  |  |  |  |  | |
|  | MR | 14 | 0.1045335 | 0.042832 | 1.110193 | 1.020796295 | 1.207417818 | 0.031126 | 0.919549 |  |  |  |  |  |  |  | |
|  | Weighted | 14 | 0.0787744 | 0.031683 | 1.08196 | 1.016815299 | 1.15127868 | 0.010327 | 0.743065 |  |  |  |  |  |  |  | |
|  | Inverse | 14 | 0.048452 | 0.02459 | 1.049645 | 1.000255851 | 1.101472735 | 0.048792 | 0.707129 | increased | -0.0174 | 0.0109 | 0.1358 | 12.24 | 0.5082 | 0.4114 | |
|  | Simple | 14 | 0.0130814 | 0.083535 | 1.013167 | 0.860150277 | 1.193405408 | 0.88315 | 0.991813 |  |  |  |  |  |  |  | |
|  | Weighted | 14 | 0.0840894 | 0.034447 | 1.087726 | 1.016711443 | 1.163700971 | 0.031483 | 0.797147 |  |  |  |  |  |  |  | |
